# Amine–Boranes as Transfer Hydrogenation and Hydrogenation Reagents: A Mechanistic Perspective

**DOI:** 10.1002/anie.202010835

**Published:** 2021-02-25

**Authors:** Samantha Lau, Danila Gasperini, Ruth L. Webster

**Affiliations:** ^1^ Department of Chemistry University of Bath Claverton Down Bath UK

**Keywords:** amine–boranes, hydrogenation, mechanisms, solvolysis, transfer hydrogenation

## Abstract

Transfer hydrogenation (TH) has historically been dominated by Meerwein–Ponndorf–Verley (MPV) reactions. However, with growing interest in amine–boranes, not least ammonia–borane (H_3_N⋅BH_3_), as potential hydrogen storage materials, these compounds have also started to emerge as an alternative reagent in TH reactions. In this Review we discuss TH chemistry using H_3_N⋅BH_3_ and their analogues (amine–boranes and metal amidoboranes) as sacrificial hydrogen donors. Three distinct pathways were considered: 1) classical TH, 2) nonclassical TH, and 3) hydrogenation. Simple experimental mechanistic probes can be employed to distinguish which pathway is operating and computational analysis can corroborate or discount mechanisms. We find that the pathway in operation can be perturbed by changing the temperature, solvent, amine–borane, or even the substrate used in the system, and subsequently assignment of the mechanism can become nontrivial.

## Graphical abstract

This Review focuses on the growing area of transfer hydrogenation (TH) using amine–boranes, looking specifically at the mechanisms by which TH takes place. There are three distinct pathways of TH: 1) classical TH process where double hydrogen transfer comes from both amine and borane; 2) nonclassical TH process where hydroboration from the amine–borane is followed by solvolysis; and 3) hydrogenation from H_2_ release.

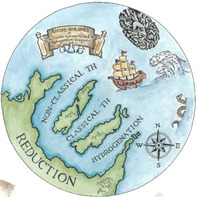

## 1 Introduction

Hydrogenation is one of the most important and fundamental transformations used in chemistry. The direct addition of dihydrogen gas (H_2_) across an unsaturated moiety is a well‐developed area of reduction chemistry which has resulted in H_2_ as the preferred hydrogen source in many of these transformations.^[1]^ However, transfer hydrogenation (TH) offers an alternative pathway that avoids the use of highly pressurized gas and potentially proffers greater control in the level of reduction. Here, a sacrificial TH agent is used to donate hydrogen, whereby the TH agent is usually cheap, abundant, and easily manipulated. Early examples include Meerwein–Ponndorf–Verley (MPV) reductions using secondary alcohols as the TH agent to reduce aldehydes and ketones to their respective alcohols.^[2]^ Furthermore, progression into asymmetric transfer hydrogenation (ATH) reactions, mediated by ruthenium catalysts, were pioneered in the 1980s using isopropanol or an azeotropic mixture of formic acid and triethylamine as the TH agent; this first report has since spawned a great interest in this area alone.^[3]^

Beyond isopropanol and formic acid, where the by‐products formed, acetone and CO_2_, respectively, are usually trivial to separate from the reaction mixture, additional TH agents reported in literature includes Hantzch esters,^[4]^ dimethylformamide with a base as additive,^[5]^ sodium hypophosphite,^[6]^ benzothiazoline,^[7]^ and hydrazine,^[8]^ although more commonly in the form of hydrazine hydrate.^[9]^ More recently, ammonia–borane (H_3_N⋅BH_3_) has been studied and shown to be a promising addition to the numerous TH agents reported to date. The low molecular weight (30.87 g mol^−1^), high hydrogen content (19.6 % wt %), and ease of handling as a bench‐stable crystalline solid makes H_3_N⋅BH_3_ an attractive TH agent. Furthermore, derivatives of H_3_N⋅BH_3_, amine–boranes (R_3−*n*_H_*n*_N⋅BH_*n*_R′_3−*n*_) and metal amidoboranes (MAB)^[10]^ have also been studied as hydrogen donors but so far are less developed in the area of TH chemistry. The advantages of using amine–boranes over H_3_N⋅BH_3_ can be found in their: 1) greater solubility in common analytical solvents such as benzene and chloroform, 2) ease of identification of by‐products in TH reactions as some amine–boranes are less likely to form insoluble polymeric substances, and 3) greater control of reduction by altering R groups on both the nitrogen and boron atom and by virtue of fewer hydrogen atoms available to transfer to the acceptor molecule.

In this Review we will present recent publications that use H_3_N⋅BH_3_ and amine–borane TH agents with an emphasis on understanding the mechanism operating in these reactions.^[11]^ Pertinently, this Review is not an evaluation of the chemistry of dehydrogenation/dehydrocoupling (DHC) of amine–boranes, for which there are numerous reviews,^[12]^ but instead focuses exclusively on the related tandem dehydrogenation TH process. Therefore, two fundamental questions present themselves in order to clarify the following discussion: What is the origin of the hydrogen atoms and how are they transferred to the substrate? We find that the literature studies reviewed here can be classed as: 1) classical TH processes whereby the double hydrogen transfer comes from both the amine and borane (Sections 2 and 3), 2) nonclassical TH processes whereby hydroboration from the amine–borane is followed by solvolysis (Section 4), and 3) hydrogenation via H_2_ released from dehydrogenation of amine–borane (Section 5). The latter process is therefore not strictly a TH process as described by Wang and Astruc: “TH reaction, referring to the addition of hydrogen to a molecule from a non‐H_2_ hydrogen source, is a convenient and powerful method to access various hydrogenated compounds”.^[13]^ However, we believe summarizing the different reduction pathways that can occur is important for a round understanding of the topic.

By targeting the mechanism of these reactions, this will aid the future design of catalytic processes using H_3_N⋅BH_3_, amine–boranes, and MAB as TH agents. Moreover, it provides a simple framework into the methodology one can apply to probe the mechanism of reduction chemistry involving amine–boranes and confirm whether classical TH, nonclassical TH, or hydrogenation mechanism is in operation.

## 2 Catalyst‐Free Classical TH of Preactivated Substrates

The TH of substrates without a catalyst has been achieved for molecules containing a polarized unsaturated bond. These reactions therefore are not applicable to a great range of substrates but still provide vital mechanistic understanding into this elementary reaction that can be informative for catalyzed processes (Section 3). In addition to lowering the activation barrier using preactivated substrates, the formation of by‐products from dimerization and cyclisation of amine–boranes could provide the entropic and enthalpic driving forces of the forward reactions. In this section we will review notable examples from the literature that have pioneered uncatalyzed TH using amine–boranes but also have an emphasis on mechanistic investigation in their studies.

**Scheme 1:**
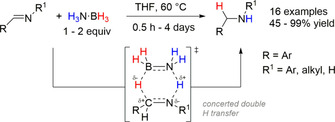
TH of imines with H_3_N⋅BH_3_ via a concerted double H transfer.

In 2010, Berke and co‐workers reported the TH of imines with 1–2 equiv of H_3_N⋅BH_3_ in tetrahydrofuran (THF) to generate the corresponding amines in good to excellent yields with concomitant formation of borazine (BZ) or polyborazylene (PBZ) as the by‐product (Scheme 1).^[14]^ The working hypothesis for this reaction was that the polarity match between the protic H_N_ and hydridic H_B_ of H_3_N⋅BH_3_ with the polarized N^δ−^=C^δ+^ moiety of the substrate would allow for spontaneous double H transfer. To probe the mechanism of this reaction and confirm this hypothesis, the reaction temperature was kept below 60 °C ensuring that no thermal decomposition of H_3_N⋅BH_3_ occurred and avoiding H_2_ release, therefore simple hydrogenation was omitted as a reaction pathway.^[15]^ Additionally, heating a mixture of H_3_N⋅BH_3_ and D_3_N⋅BD_3_ at 60 °C in THF resulted in no deuterium scrambling, indicating that the adduct does not dissociate and therefore Lewis acid (BH_3_) or base mediated (NH_3_) transfer hydrogenation could also be discounted.

Deuterium labeling experiments were performed using benzylidene aniline as the model substrate. Using H_3_N⋅BD_3_, deuterium incorporation was found solely at the C atom of the imine moiety; conversely, using D_3_N⋅BH_3_ resulted in deuterium incorporation exclusively at the N atom. Using D_3_N⋅BD_3_ gave deuterium incorporation both at the C and N atoms. These experiments corroborated the polarity match mechanism, which was found to be feasible based on quantum mechanics calculations performed. A six‐membered transition state (TS) whereby the double H transfer of N−H⋅⋅⋅N and the B−H⋅⋅⋅C was found to be 23.5 kcal mol^−1^ more favorable than the polarity mismatch configuration. In order to decipher whether the mechanism was via a concerted or stepwise process, primary deuterium kinetic isotope effect (DKIE) experiments were undertaken, revealing: 1) inverse DKIE (0.87) when H_3_N⋅BD_3_ was used, 2) a normal DKIE (1.93) when D_3_N⋅BH_3_ was used, and 3) a small positive DKIE (1.39) when D_3_N⋅BD_3_ was used. Hammett correlations also revealed positive values of the sensitivity constants (*ρ*) for *para*‐substituted benzylidene anilines (substitution at aniline side, *ρ*=1.61 and substitution at benzylidene side, *ρ=*0.69). All these results indicate an asynchronous concerted double H transfer, whereby the breaking of the N−H bond was the rate‐determining step (RDS) of the transformation.

**Scheme 2:**
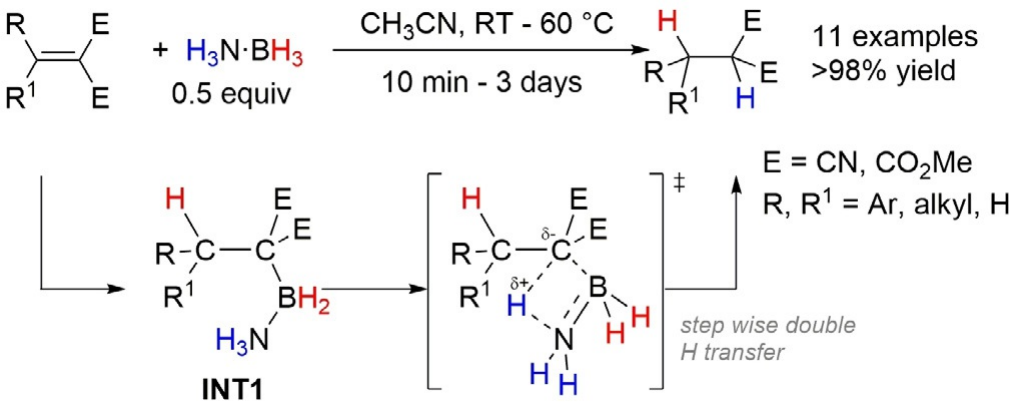
TH of polarized olefins via stepwise double H transfer.

In related studies, Berke and co‐workers expanded this methodology for polarized olefins (Scheme 2).^[16]^ The reactions were too quick to be monitored by NMR spectroscopy in THF, so acetonitrile was used as slower reactivity was observed in this solvent. Deuterium labeling experiments confirmed the polarity match of the hydridic H_B_ and protic H_N_ transfer to the C atom with aryl/alky groups and C atom with electron‐withdrawing substituents, respectively. However, the measured DKIE using 2‐cyclohexylidenemalononitrile as the model substrate revealed 1) no DKIE (1.00) when H_3_N⋅BD_3_ was used, 2) a normal DKIE (1.55) when D_3_N⋅BH_3_ was used, and 3) a normal DKIE (1.61) when D_3_N⋅BD_3_ was used. This indicated that this was a stepwise process whereby the RDS involved cleavage of the N−H bond. Further experimentation of a 1:3 mixture of H_3_N⋅BH_3_ with 2‐cyclohexylidenemalononitrile at −40 °C in [D_8_]THF (and also in CD_3_CN) allowed the authors to observe the hydroboration intermediate **INT1** by in situ multinuclear NMR spectroscopy, suggesting the mechanism involved a facile hydroboration step preceding the RDS. This mechanism was distinctly different to that observed with imines (vide supra). Continuing on from this work, Berke and co‐workers were also able to effect the TH of polarized olefins using MeH_2_N⋅BH_3_, *t*BuH_2_N⋅BH_3_, and Me_2_HN⋅BH_3_ as well as H_3_N⋅BH_3_ as the hydrogen donor.^[17]^ Mechanistic investigation showed similar results of a stepwise double H transfer.

**Scheme 3:**
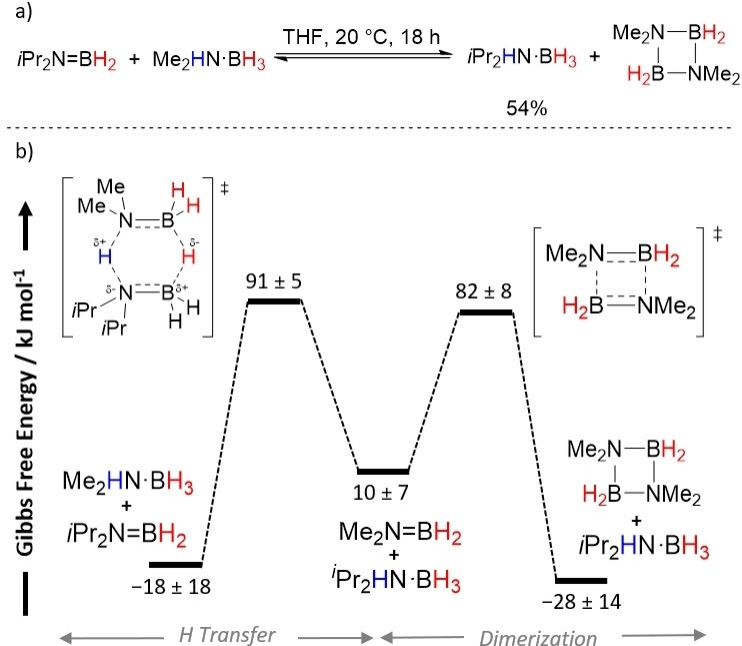
a) Reversible TH of *i*Pr_2_N=BH_2_ with Me_2_HN⋅BH_3_ and b) simplified reaction profile of the forward reaction.^[18]^

In 2011, Manners and co‐workers reported the metal‐free TH between several amine–boranes and the aminoborane *i*Pr_2_N=BH_2_ in THF at 20 °C (Scheme 3 a).^[18]^ Experimental and computational investigation into this reaction followed.^[19]^ Me_2_HN⋅BH_3_ was chosen as the model substrate in these studies. Experimentally the reaction proceeded more cleanly than the reaction with MeH_2_N⋅BH_3_ and H_3_N⋅BH_3_, with the only side‐product being cyclodiborazane [Me_2_N–BH_2_]_2_. However, the reaction reached equilibrium at ≈50 % conversion.

Overall, a mechanism analogous to that reported by Berke for the TH of imines^[14]^ was proposed, involving an asynchronous concerted double H transfer (Scheme 3 b). However, the thermodynamic accessibility of the two reactions was vastly different. When the TH of *i*Pr_2_N=BH_2_ with MeH_2_N⋅BH_3_ was monitored by multinuclear NMR spectroscopy at varying temperatures, the calculated thermodynamic parameters showed the TH from Me_2_HN⋅BH_3_ to *i*Pr_2_N=BH_2_ was endergonic (Δ*G*°_(295)_=10±7 kJ mol^−1^) but the dimerization of the transient [Me_2_N=BH_2_] species was more exergonic (Δ*G*°_(295)_=−28±14 kJ mol^−1^) and therefore driving the reaction in the forward direction. The measured large entropy and small enthalpy of activation for the forward TH reaction (Δ*S* ^≠^_(295)_=−210±11 kJ mol^−1^ and Δ*H* ^≠^_(295)_=29±5 kJ mol^−1^) were consistent with a highly ordered bimolecular TS, suggesting a concerted TS with values similar to those previously reported for Diels–Alder reactions.^[20]^ DKIE experiments with *i*Pr_2_N=BH_2_ showed a large positive DKIE (*k*_H/_ *k*_D_=6.7±0.9) when Me_2_DN⋅BH_3_ was used, but a small inverse DKIE (*k*_H/_ *k*_D_=0.7±0.1) with Me_2_HN⋅BD_3_ and a large positive DKIE (*k*_H/_ *k*_D_=5.2±0.8) with Me_2_DN⋅BD_3_. Manners and co‐workers rationalized the small inverse DKIE obtained for the hydride transfer as the result of a secondary kinetic isotope effect and the change in the geometry around the boron atom at the TS. Density functional theory (DFT) calculations of these thermodynamic parameters gave a good match to the experimental values. Furthermore, DFT calculations showed that alternative pathways, such as stepwise addition with B−H⋅⋅⋅B transfer first, stepwise addition with N−H⋅⋅⋅N transfer first, or a dissociative process were energetically unfeasible and did not align with the experimental evidence.

It is worth noting that this study focused on Me_2_HN⋅BH_3_ as the TH partner. However, when RR′HN⋅BH_3_ was used (where R=H and R′=Me or H), an additional by‐product was observed: [H_2_B(μ‐H)(μ‐NRR′)BH_2_]. This would suggest that under these reaction conditions a stepwise or dissociative pathway could be operating,^[19]^ and highlights the sensitivity of these reaction pathways and how they could be perturbed by simply changing the substituents on the amine–boranes used.

More recently, Braunschweig and co‐workers reported the TH of three iminoboranes with bulky R substituents (R‐N≡B‐R^1^, where R=*t*Bu and R^1^=*t*Bu, mesityl, or 2,3,5,6‐tetramethylphenyl) with H_3_N⋅BH_3_.^[21]^ They calculated that the formation of two aminoboranes as the products (more accurately the cyclization products from H_2_N=BH_2_) would be thermodynamically favorable to drive the reaction forward. Placing *t*Bu‐N≡B‐*t*Bu under high H_2_ pressure led to no hydrogenation even in the presence of Pd/C catalyst, indicative that a classical TH process was occurring. Although the multinuclear NMR and FTIR spectroscopic data supported the formation of *t*BuHN=B*t*BuH as the product, the *cis*/*trans* stereochemistry of this aminoborane was not clear. Isolation of the products for X‐ray diffraction analysis from the subsequent reaction of *t*BuHN=B*t*BuH with HCl or the N‐heterocyclic carbene 1,3‐diisopropylimidizol‐2‐ylidene (IPr) suggested the aminoborane carried *trans* stereochemistry.

**Scheme 4:**
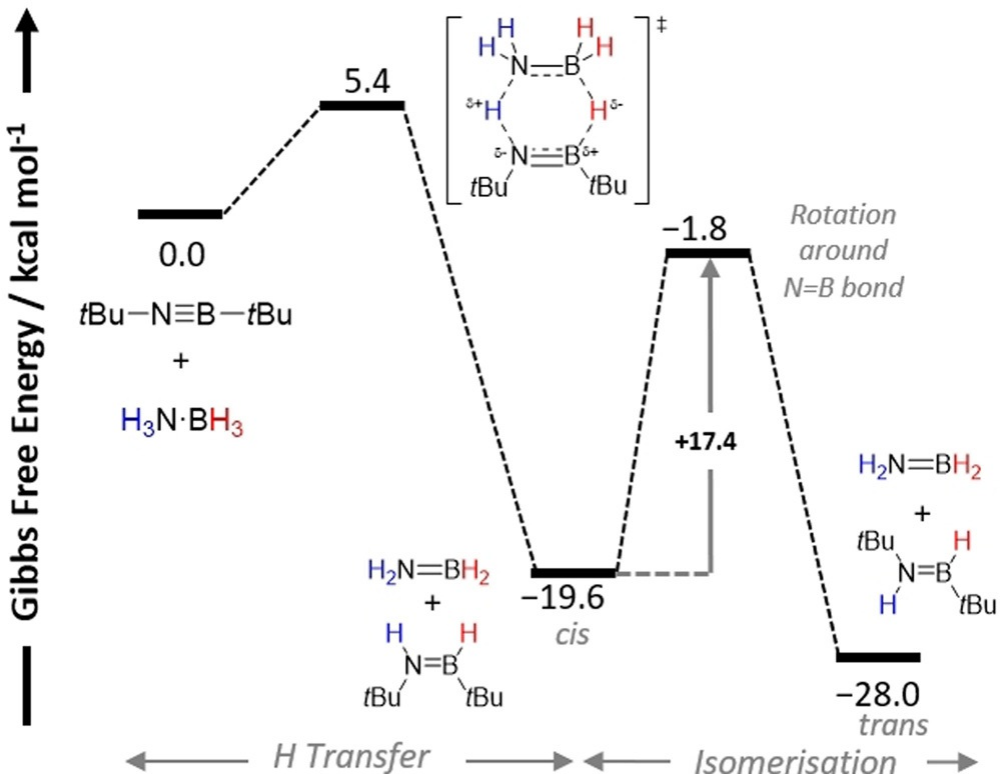
Simplified reaction profile of TH of *t*Bu‐N≡B‐*t*Bu with H_3_N⋅BH_3_.^[21]^

Probing the mechanism further, deuterium labeling experiments using D_3_N⋅BH_3_ and H_3_N⋅BD_3_ confirmed the polarity matching of the substrates. However, no DKIE experiments were reported to substantiate the DFT calculations, which supported a concerted double H transfer through a very low‐energy TS (5.4 kcal mol^−1^) (Scheme 4). This concerted addition would lead to the *cis*‐aminoborane, which was 8.4 kcal mol^−1^ higher in energy than the *trans*‐aminoborane. Observation of the c*is* conformation would align with the proposed mechanism, but the *trans*‐aminoborane as the final product was inferred experimentally (vide supra) and could indicate a stepwise pathway instead. However, a rotation around the N=B bond to allow the isomerization from *cis* to *trans* was found through a relatively high barrier of 17.8 kcal mol^−1^ at room temperature. This isomerization step would therefore be the RDS and in theory the *cis*‐aminoborane should be observed by in situ multinuclear NMR spectroscopy prior to isomerization, but this was not reported by the authors. This could indicate that an alternative isomerization pathway with a much lower barrier could be involved than the one calculated and reported; low‐temperature studies could give insight.

**Scheme 5:**
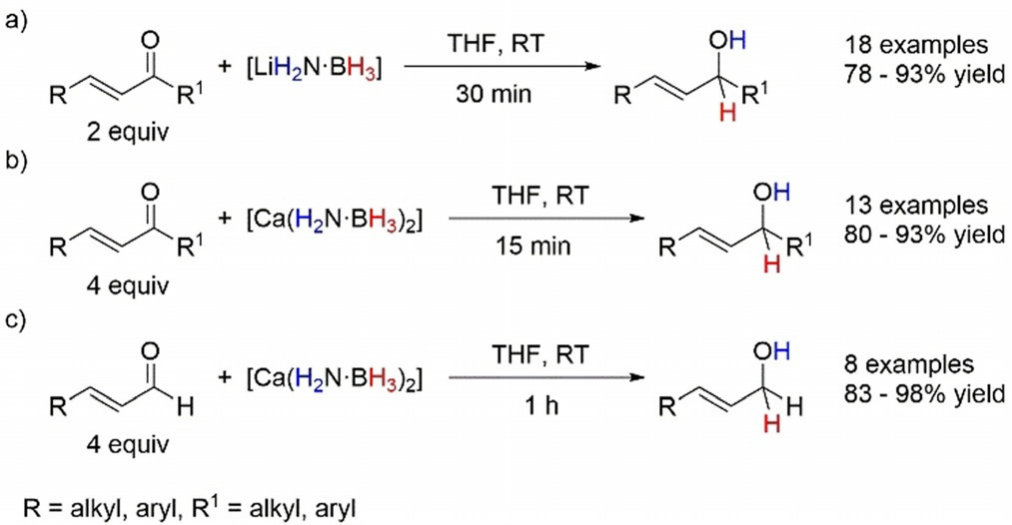
TH of α,β‐unsaturated ketones and aldehydes to allylic alcohols by MABs.

In 2012, Chen and co‐workers reported a number of studies using lithium amidoborane (LiH_2_N⋅BH_3_) and calcium amidoborane (Ca(H_2_N⋅BH_3_)_2_) to chemoselectively reduce α,β‐unsaturated ketones to allylic alcohols under ambient temperature (Scheme 5 a,b).[^10c^, ^22^] Using MABs circumvented the use of conventional reducing agents such as NaBH_4_, which often has poor selectivity from over reduction of the substrate, or using lithium aminoborohydrides (LiR_2_N⋅BH_3_, where R≠H), which requires a subsequent hydrolysis step. Optimization of the reaction found THF to be the best solvent, as MeOH resulted in solvolysis of the MAB. Keeping the reactions at ambient temperature negated dehydrogenation of the MAB, with these processes occurring at elevated temperatures (LiH_2_N⋅BH_3_, ≈40 °C;^[23]^ Ca(H_2_N⋅BH_3_)_2_, ≈80 °C^[24]^). Deuterium labeling experiments using [M^*n*+^(D_2_N⋅BH_3_)_*n*_^−^] (M=Li or Ca) showed deuterium incorporation only at the oxygen, and when [M^*n*+^(H_2_N⋅BD_3_)_*n*_^−^] was used only at the C atom of the carbonyl moiety, confirming the double H transfer process and that the hydrogen came from the respective MAB.

A further comparison of the two different MABs revealed that Ca(H_2_N⋅BH_3_)_2_ was more competent at the TH of α,β‐unsaturated aldehydes to the allylic alcohol (Scheme 5 c). When the Chen group reacted LiH_2_N⋅BH_3_ with the substrate, they observed full conversion of the starting material but only ≈50 % of the allylic product was formed according to ^1^H NMR spectroscopy. A white precipitate observed in the reaction mixture was assigned to a lithium aminoarylborate species, which upon hydrolysis with aqueous HCl gave the desired alcohol. This lower reactivity was postulated as a result of poor solubility of the intermediate in THF and the potentially higher enthalpic penalty of losing a second (Li)N−H bond versus (Ca)N−H bond.[^10b^, ^10c^]

Chen and co‐workers followed up this work by reporting the TH of ketones and imines with LiH_2_N⋅BH_3_, Ca(H_2_N⋅BH_3_)_2_, and also sodium amidoboranes (Na(H_2_N⋅BH_3_) with high conversion to secondary alcohols and amines, respectively, across all MABs used.^[25]^ Higher reactivities were displayed by the MABs in comparison to H_3_N⋅BH_3_ in these TH reactions. This was attributed to the weaker B−H bond of the former due to a more electron‐rich B center^[10a]^ and also M⋅⋅⋅H−B interactions.^[26]^ The reaction mechanism of these TH reaction kinetic studies was probed using LiH_2_N⋅BH_3_ with benzophenone and *N*‐benzylideneaniline, and a first order dependence with respect to LiAB and a zeroth order dependence on the substrate was found. Additional DKIE experiments carried out using benzophenone revealed a small positive DKIE (1.26) with LiD_2_N⋅BH_3_ and a larger positive DKIE (1.89) with LiH_2_N⋅BD_3_. Similar values were obtained when *N*‐benzylideneaniline was used (LiD_2_N⋅BH_3_: 1.26; LiH_2_N⋅BD_3_: 2.12). From these experiments they proposed that the B−H bond breaking is involved in the RDS.

**Scheme 6:**
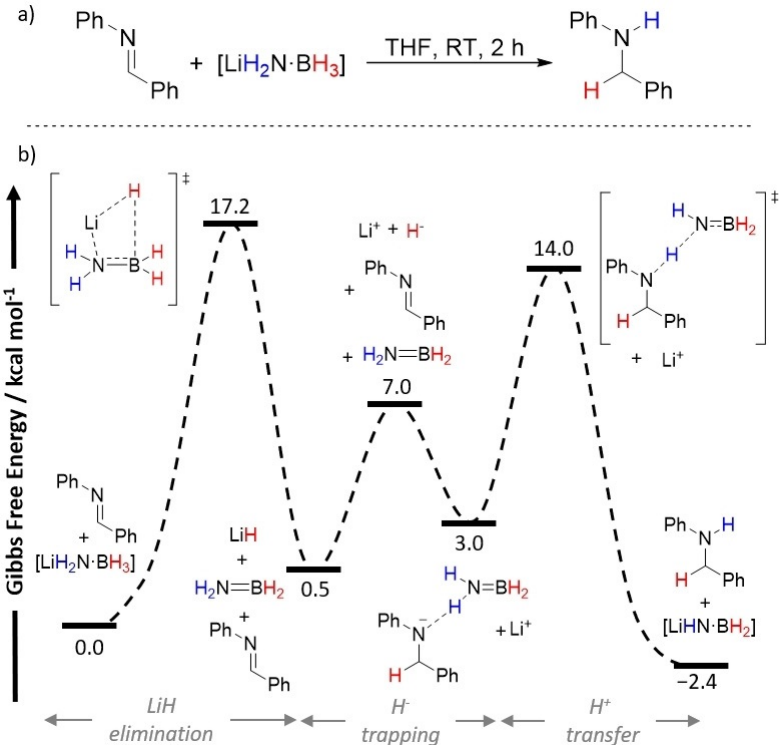
a) TH of *N*‐benzylideneaniline with LiH_2_N⋅BH_2_; b) simplified reaction profile of the TH reaction.^[25]^

Although MABs were reported to be superior TH agents than H_3_N⋅BH_3_ in these studies, the addition of the alkali and alkaline earth metals complicates the mechanism operating in these reactions. Chen et al. reported a complementary DFT investigation of the TH of *N*‐benzylideneaniline with LiH_2_N⋅BH_3_ (Scheme 6).^[25]^ A calculated pathway was found which involved the initial elimination of LiH from LiH_2_N⋅BH_3_, representing the RDS of the reaction at Δ*G* ^≠^=17.2 kcal mol^−1^. This RDS agreed with the kinetic and DKIE experiment showing zeroth order dependence on the substrate *and* the breaking of the B−H bond in this step. Chen et al. also attributed the small DKIE observed when LiD_2_N⋅BH_3_ was used as a consequence of the small difference in energy between the two TSs, ΔΔ*G* ^≠^=3.2 kcal mol^−1^, involving both the N−H and B−H bond‐breaking steps. Furthermore, they found a higher RDS (Δ*G* ^≠^=28.0 kcal mol^−1^) when H_3_N⋅BH_3_ was used as the TH agent which matched the higher reactivity displayed by LiH_2_N⋅BH_3_ in the reduction reactions.

## 3 Catalyzed Classical TH Reactions

In this section we highlight the chronological development of catalyzed, along with some stoichiometric, TH reactions where amine–boranes are required as hydrogen source and precatalyst activator; it is vital to comprehend that in a classical TH the amine–borane assumes this double role, allowing the formation of an active species/catalyst and also furnishing the H^+^/H^−^ critical to reduction. Classifying the following reactions as classical TH therefore makes it possible to distinguish them from nonclassical TH (Section 4) and hydrogenation reactions (Section 5). Following the aim of this Review, the focus will be given to studies where the mechanistic investigations are detailed. It is worth noticing that some reactions cannot be classified exactly in the three main categories that we have chosen to investigate, and that grey areas exist with mechanistic changes occurring with varying substrates and/or reaction conditions. Therefore, we carefully comment and propose a rationale for these unclear points to allow a complete description of the topic and provide a thoughtful analysis of classical TH with amine–boranes.

### 3.1 Metal Catalysis

**Scheme 7:**
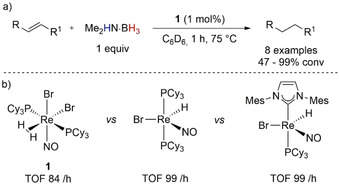
a) General TH of olefins with Me_2_HN⋅BH_3_ and with Re^I^ precatalyst **1**; b) range of Re^I^ precatalysts and TOF results for the TH of octene after 1 h reaction at 85 °C.

One of the first examples of metal‐catalyzed transfer hydrogenation of olefins with amine–boranes was reported by Berke and co‐workers (Scheme 7).^[27]^ The authors used 1 mol % of [Re(Br)_2_(NO)(PCy_3_)_2_(η^2^‐H_2_)] **1** to convert octene into octane with Me_2_HN⋅BH_3_. The reaction allowed quantitative conversion in 1 h, irrespective of whether the reaction was carried out in an open or closed vessel, suggesting that H_2_ was not responsible for the reduction. Initial stoichiometric studies hinted to the importance of transient phosphine dissociation from the metal precursor to allow oxidative addition of the amine–borane to Re^I^, with an excess of phosphine found to decrease reactivity drastically. The reaction mechanism proceeded stepwise, with initial B−H σ‐bond activation and oxidative addition to form a Re^III^ species, followed by hydride insertion and Re–alkyl bond formation. After a β‐hydride shift to liberate cycloborazine, the reductive elimination step ensured product formation and catalyst regeneration.

The authors followed up on the TH with Me_2_HN⋅BH_3_ by using the five‐coordinate [Re(Br)(NO)(PCy_3_)(H)(L)] (L=1,3‐dimesitylimidazol‐2‐ylidene IMes or PCy_3_) precatalyst, which allowed a slight increase in the reaction efficiency (Scheme 13 b).^[27b]^ Subsequently the Re‐catalyzed TH reaction was applied to the reduction of terminal olefins,^[28]^ relying, however, on the ethanolysis of amine–boranes (Section 4).

**Scheme 8:**
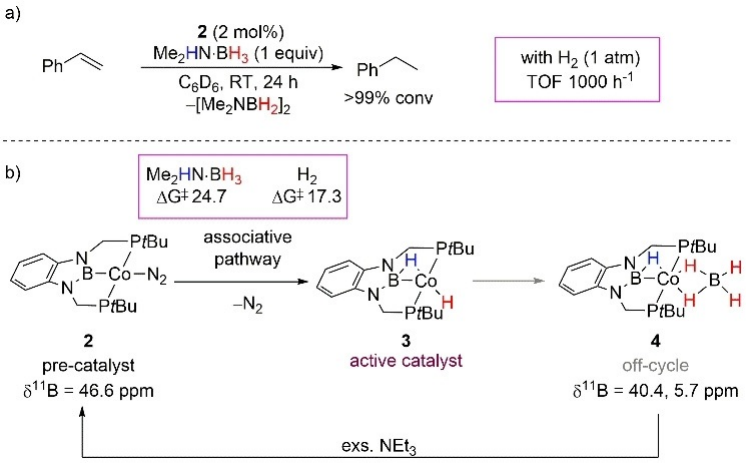
a) General scheme of the Co^I^‐catalyzed hydrogenation of styrene with Me_2_HN⋅BH_3_; b) major reaction intermediates with details on the RDS for active catalyst formation (kcal mol^−1^).

In 2013, Lin and Peters explored the reduction of olefins by Co–boryl complex **2** (Scheme 8 a);^[29]^ the formation of a Co–hydridoborane complex **4** was found when **2** was subjected to an excess of Me_2_HN⋅BH_3_ (Scheme 8 b), and the structure was confirmed by X‐ray analysis and NMR spectroscopy. It was noted that the reduction of styrene was dramatically faster under an atmosphere of H_2_ with full conversion to ethylbenzene in 1 h versus the 24 h needed when Me_2_HN⋅BH_3_ was used. Paul and co‐workers analyzed the computational details of this reduction;^[30]^ the authors calculated that in the presence of amine–borane, complex **2** can form **3** via an associative mechanism with an activation energy of 24.7 kcal mol^−1^. However, the generation of active catalyst **3** was found to be more energetically demanding with Me_2_HN⋅BH_3_ than its formation in the presence of molecular H_2_ (17.3 kcal mol^−1^), which confirms the results observed experimentally. Co–hydridodiborane **4**, which formed only in the presence of amine–borane, was found to be off‐cycle and was described as resulting from the decomposition of **3**. Further experimental and computational details from Paul and co‐workers showed that an excess of base NEt_3_ can convert **4** back to **2**.

**Scheme 9:**
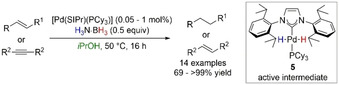
NHC‐Pd catalyzed TH of alkenes and alkynes with H_3_N⋅BH_3._

Studies from Cazin and co‐workers showed an efficient [Pd(NHC)(PR_3_)]‐catalyzed TH of alkenes and alkynes with H_3_N⋅BH_3_ (Scheme 9).^[31]^ The active intermediate [Pd(H)_2_(IPr)(PCy_3_)] **5** was isolated,^[32]^ and its formation and role in the hydrogenation was computationally clarified by Yi and co‐workers.^[33]^ Intermediate **5** formed via sequential ligand‐assisted N–H followed by B–H activation of H_3_N⋅BH_3_ (Δ*G* ^≠^=23.8 kcal mol^−1^). Stepwise TH from **5**, instead of molecular H_2_, was found to be kinetically and thermodynamic favorable with an energy barrier of 22.3 kcal mol^−1^, thus highlighting TH *not* hydrogenation is in place in this Pd‐catalyzed system. The role of *i*PrOH in the reaction mechanism was not analyzed in detail, thus the possibility of solvolysis for this TH process cannot be ruled out (Section 4.).

A nickel version of alkyne TH was performed by Garcia and Barrios‐Francisco using [Ni(dppe)(η^2^‐dpa)] (dpa=diphenylacetylene); the authors showed the selective semi‐TH of alkynes with H_3_N⋅BH_3_.^[34]^ The observed stereoselective divergency to *cis* or *trans* olefin was dependent on the solvent, with THF favoring the former while MeOH furnished *trans* selection. It is worth highlighting that a nonclassical TH might be in action when polar protic solvent is used, following our considerations on solvolysis‐mediated TH reactions (Section 4). No trace of H_2_ was evident by GC‐MS in the catalytic tests, which highlights that the TH mechanism might be the preferred pathway for this transformation.

The first example of semi‐TH of alkynes with H_3_N⋅BH_3_ in the presence of copper was reported in 2017;^[35]^ the authors used air‐stable [Cu(IPr)(OH)] and an excess of H_3_N⋅BH_3_ to selectively reduce alkynes to (*Z*)‐alkenes in THF at 50 °C, and further expanded the substrate scope to the full reduction of propiolates. A blank reaction under 1 bar of H_2_ with a catalytic amount of H_3_N⋅BH_3_ (20 mol %) allowed only 20 % conversion into product, which suggested a direct hydride transfer mechanism to be in place.

**Scheme 10:**
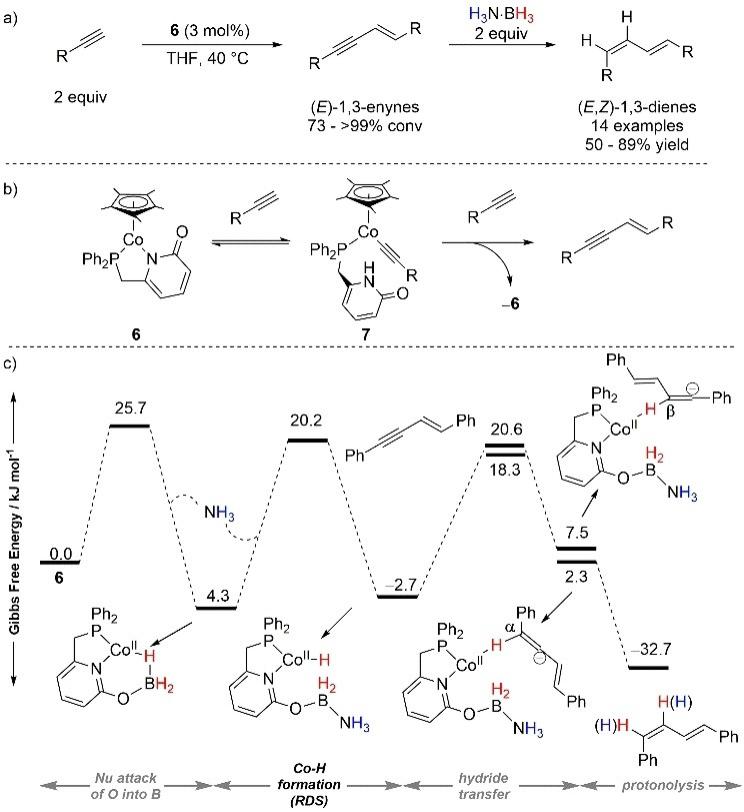
a) Sequential dimerization/semi‐TH of alkynes catalyzed by PN–Co^II^ complex **6**; b) details of dimerization mechanism; c) Gibbs free energy diagram for the TH reaction of 1,3‐enynes into 1,3‐dienes.

Wang, Liao, and co‐workers reported an elegant sequential dimerization/semihydrogenation reaction of alkynes into (*E*,*Z*)‐1,3‐dienes with Co^II^ complex **6** and H_3_N⋅BH_3_ (Scheme 10 a).^[36]^ The authors distinguished the two sequential catalytic cycles and studied the full reaction profile both experimentally and by DFT analysis. Initial dimerization of the alkyne to a 1,3‐enyne proceeded through metal–ligand cooperative activation of the alkyne by **6** to form an alkylidene–Co complex **7** (Scheme 10 b); the latter could further react through an anti‐Markovnikov addition to a second equivalent of alkyne, release the 1,3‐enyne, and reform precatalyst **7**. The subsequent addition of H_3_N⋅BH_3_ to the reaction mixture allowed the pyridonate ligand mediated formation of a [Co–H] intermediate (Scheme 10 c); this step was proposed to proceed via borane activation by nucleophilic attack of the pyridonate ligand with ammonia release (Δ*G* ^≠^=22.1 kcal mol^−1^), which further re‐enters the cycle and attacks the new borate formed to allow Co−H bond formation (RDS with Δ*G* ^≠^=23.9 kcal mol^−1^). The latter hydride species further reacts with the enyne for the transfer reduction; the hydride transfer to the α‐carbon versus the β‐carbon atoms of the enyne differed by 0.5 kcal mol^−1^, which did not allow to distinguish the fate of H^−^ incorporation. This hypothesis was further corroborated by deuterium labeling studies, which showed that there was no distinction between deuterium incorporation in the 1,3‐diene with H_3_N⋅BD_3_ or D_3_N⋅BH_3_. Final facile intramolecular protonolysis by the amino group releases the product with a *cis* configuration of the reduced triple bond.

**Scheme 11:**
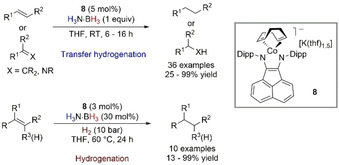
TH and hydrogenation of di‐, tri‐, and tetrasubstituted alkenes and imines catalyzed by α‐diimine cobaltate anions.

Another example of TH and hydrogenation of olefins was reported by Wolf and co‐workers.^[37]^ the authors used a catalytic amount of a reduced cobaltate anion [K(thf)_1.5_{(^IPr^BIAN)Co(COD)}] (BIAN=bis(iminoacenaphthene)diamine) to perform dehydrogenation of H_3_N⋅BH_3_, TH of disubstituted olefins and imines with H_3_N⋅BH_3_ and hydrogenation of tri‐ and tetrasubstituted alkenes (Scheme 11).

**Scheme 12:**
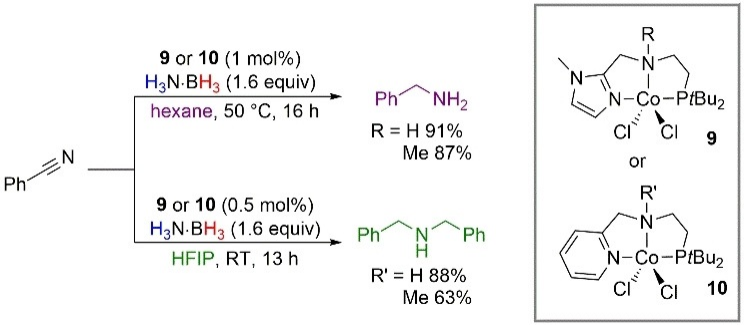
NNP–Co‐catalyzed chemoselective TH of nitriles with H_3_N⋅BH_3_.

The kinetic profiles for the dehydrogenation reaction showed a second order rate in catalyst **8** (Scheme 12), suggesting the formation of a dinuclear [Co–H] active species, with catalyst deactivation observed at higher conversions; Hg drop test and P(OMe)_3_ poisoning experiment did not affect conversions. However, when the strongly coordinating ligand dibenzo[*a*,*e*]cyclooctatetraene (dct) was used, the reaction slowed down and traces of dct partial hydrogenation were found, which suggested the process to be homogeneous. Moreover, an induction period was observed at low catalyst loading that was found to be related to partial hydrogenation of 1,5‐cyclooctadiene through poisoning experiments. The mechanism for TH was derived from these initial findings, with an observed reaction rate similar to that of the dehydrogenation reaction, which highlights that the dinuclear [Co–H] species is the common species formed in both the TH and dehydrogenation reactions. When TH of α‐methylstyrene was performed in a D_2_ atmosphere (1.1 bar), 20 % of deuterium incorporation into the cumene product was found, which supported a H_3_N⋅BH_3_‐mediated reduction. In contrast, when bulky olefins were subjected to the optimized reaction conditions, 10 bar of H_2_ was necessary to ensure product formation. This highlights that hydrogenation is in action with hindered substrates.

Liu et al. presented a catalyzed TH of nitriles by H_2_N⋅BH_3_ (Scheme 12).^[38]^ The NNP‐type cobalt pincer complexes **9** and **10** were active for a chemodivergent nitrile hydrogenation to primary, secondary, or tertiary amines, depending on the solvent. When hexane was used and benzonitrile was subjected to the reaction conditions, benzylamine was found to be the predominant product, while switching to hexafluoro‐2‐propanol (HFIP) led to the selective formation of secondary dibenzylamine. The reaction worked well with N–H or N–Me complexes, which highlights that an outer‐sphere mechanism with ligand cooperativity might not be involved in this reaction, while an inner‐sphere mechanism should be considered. Moreover, the involvement of HFIP should not be discarded, as the authors reported when studying olefin TH (Section 4.2).^[39]^

**Scheme 13:**
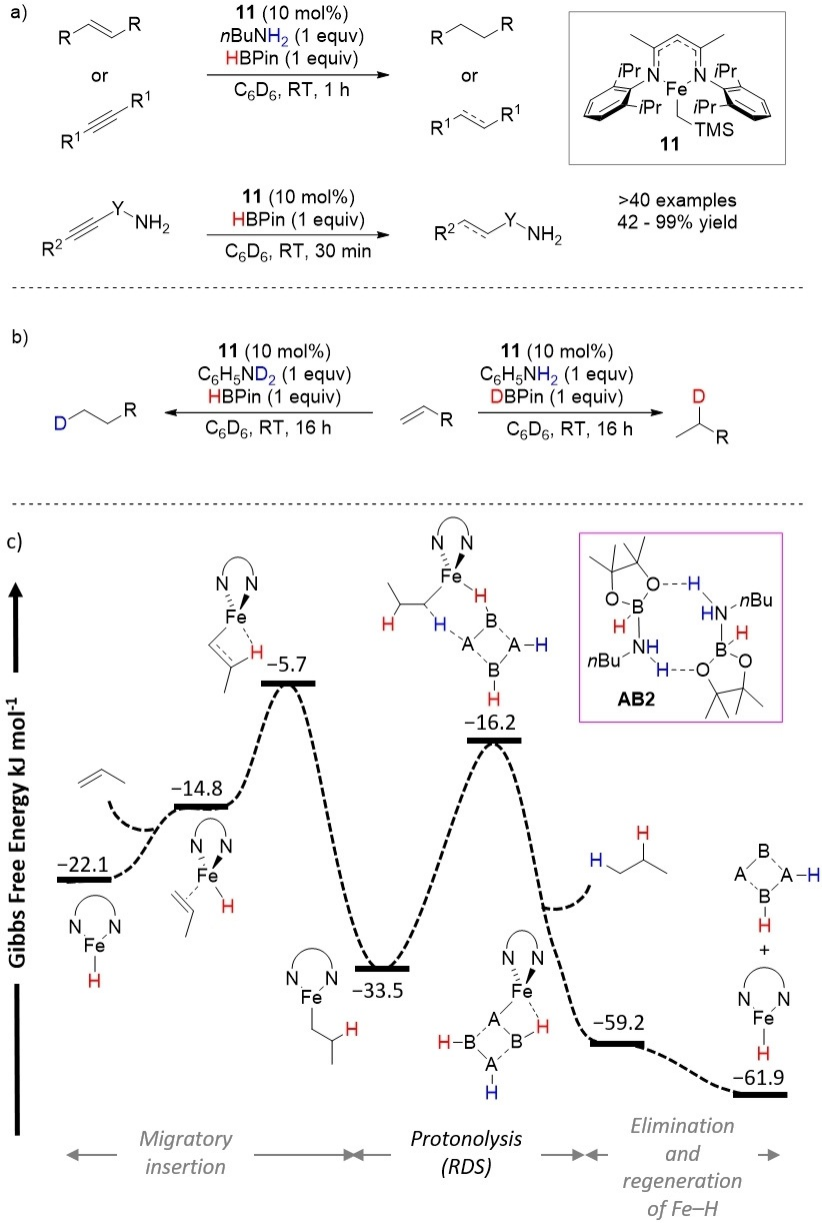
a) Fe^II^‐catalyzed TH of alkenes and alkynes; b) selective deuterium incorporation with labeled aniline or HBpin; c) details of the computed free energy profiles (kcal mol^−1^) for the competing DHC of **AB2**, preferred quintet spin state shown.

Webster and co‐workers presented a rare example of Fe^II^‐catalyzed TH and semi‐TH of olefins.^[40]^ The β‐diketiminate iron alkyl precursor **11** in the presence of sacrificial amines and borane selectively reduced alkenes and alkynes (Scheme 13 a). The catalytic system was found to be extremely robust when amino substituents were included in the substrate, by eliminating the use of a sacrificial external amine. Labeling experiments showed that selective anti‐Markovnikov monodeuteration occurred with deuterated aniline, while incorporation of deuterium was found to be predominant in the internal positions (Markovnikov product) when DBpin was used (Scheme 13 b).

Gelation was observed when the TH of olefins was performed, which, together with the lack of competitive hydroboration of substrates, prompted the authors to analyze the involvement of oligomeric species in the reaction mechanism. The formation of dimeric and tetrameric species of [*n*BuH_2_N⋅BHPin]_*x*_ (*x=*1: **AB2**; *x=*2: **AB4**) was proven based on the evident shift of the B–H signal versus free pinacol borane in the ^11^B NMR spectrum; these species were found to be entropically favored compared to DHC adducts. Subsequent DFT calculation of the catalytic cycle highlighted the formation of an [Fe–H] active species and 1,2‐alkene insertion followed by rate‐limiting protonolysis with a Δ*G* ^≠^=17.3 kcal mol^−1^ (Scheme 13 c). Therefore, the formation of oligomers in this transformation was found to be crucial to decrease the concentration of free borane and amine in solution and to allow the TH process to be the preferred pathway.

Punji and Sharma studied the TH of nitriles to secondary amines using [Co(Xantphos)Cl_2_].^[41]^ The transformation was found to be dependent on the amine–borane used; with H_3_N⋅BH_3_, the selective formation of symmetrical secondary amine was observed. When Me_2_HN⋅BH_3_ was used and the reaction was performed in the presence of a second equivalent of amine, the synthesis of unsymmetrical amines resulted instead.

**Scheme 14:**
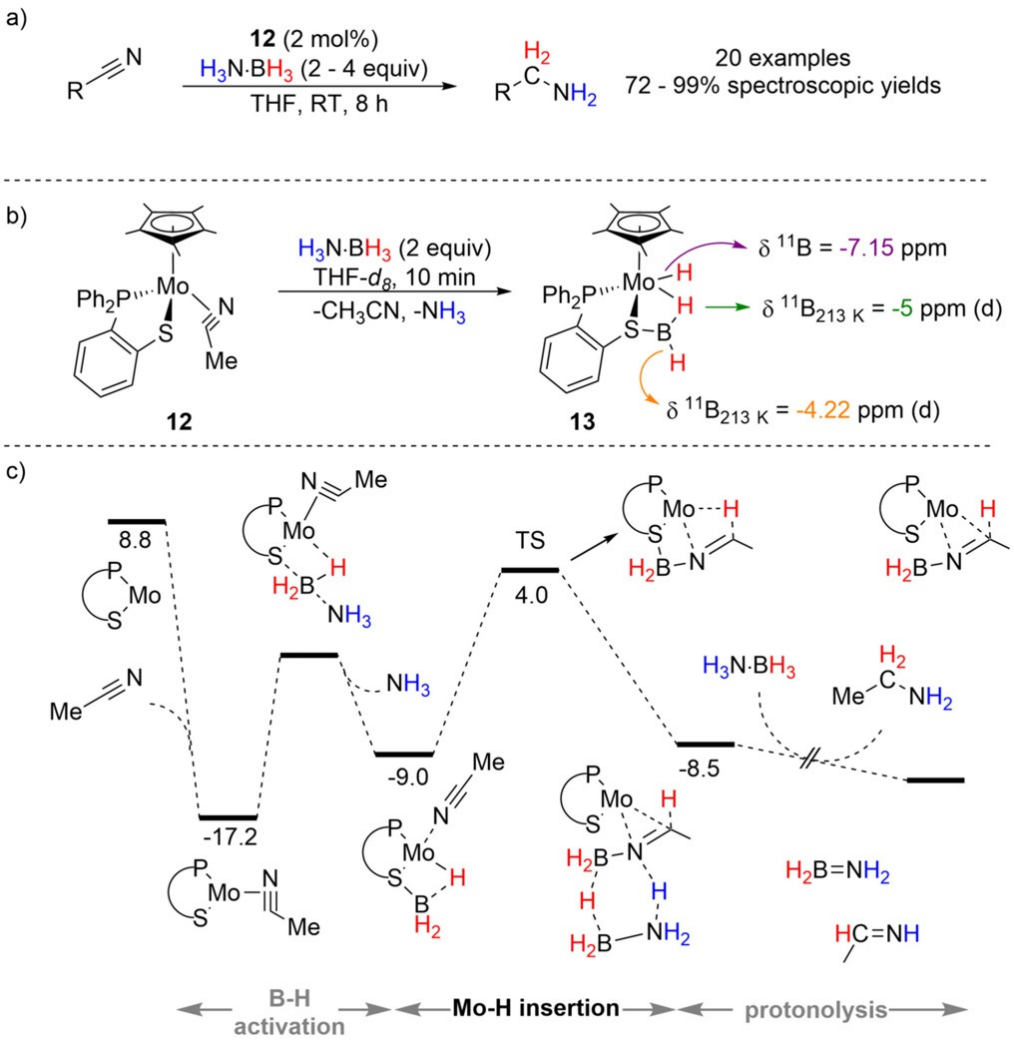
a) Molybdenum–thiolate catalyzed TH of nitriles; b) formation of the [Mo–H]/borohydride mixed species **13** and details regarding its ^11^B NMR chemical shifts; c) details of the Gibbs free energy diagram (kcal mol^−1^) of the catalytic cycle.

**Scheme 15:**
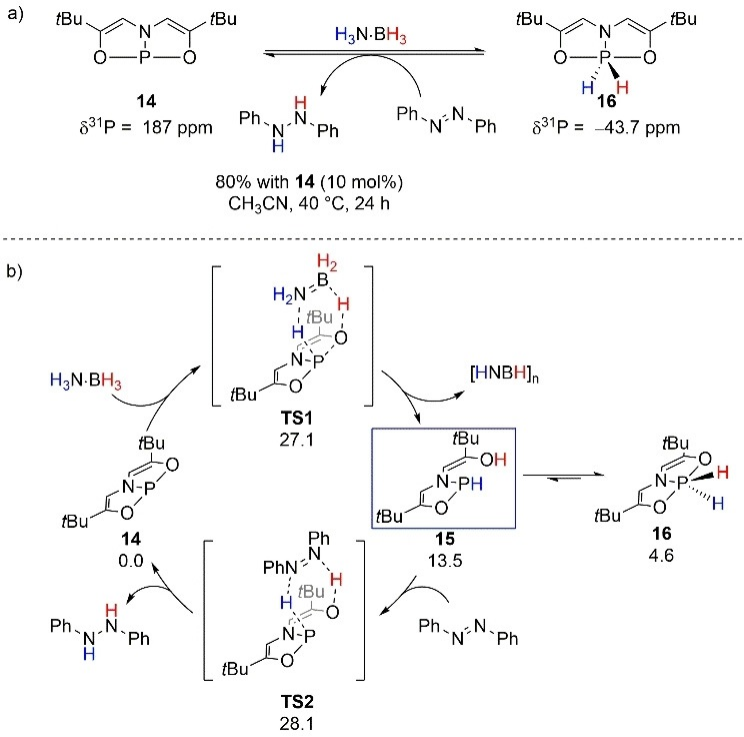
a) Initial finding for the catalyzed reduction of azoarenes with **14** using H_3_N⋅BH_3_ as the hydrogen source and the proposed formation of P^V^–hydride **16**; b) revised mechanistic study highlighting P–ONO ligand cooperativity.

Wang, Liao, and co‐workers also described a catalytic TH of nitriles using a molybdenum–thiolate complex to synthesize primary amines in the presence of H_3_N⋅BH_3_ (Scheme 14 a).^[42]^ The authors used [Cp*Mo(1,2‐Ph_2_PC_6_H_4_S)(η^2^‐NCMe)] which promptly reacts with H_3_N⋅BH_3_, activating the B−H bond via a metal–ligand cooperative action and forming a neutral Mo^II^‐H/borohydride species (**13**). The mixed species **13** was analyzed by variable‐temperature ^11^B NMR spectroscopy, which showed that a fast interchange was in effect between the Mo‐H‐B and the B−H bond at room temperature (Scheme 15 b). The latter species **13** could catalyze the TH of nitriles but only in the presence of added H_3_N⋅BH_3_. Kinetic studies showed a first order dependence in H_3_N⋅BH_3_ and precatalyst **12**, while the reaction was zeroth order in substrates, which ruled out nitrile activation as the RDS. Further DKIE values were determined, (*k* ${}_{D{}_{3}N\cdot \mathrm{BH}{}_{3}}$ =1.4, *k* ${}_{H{}_{3}N\cdot \mathrm{BD}{}_{3}}$ =2.6, and *k* ${}_{D{}_{3}N\cdot \mathrm{BD}{}_{3}}$ =3.3) which could suggest that both N−H and B−H bond activation are involved in the RDS. However, further computational calculations to describe the catalytic cycle indicate that, instead, Mo–H insertion into the C≡N bond with a Δ*G* ^≠^ of 22 kcal mol^−1^ is the RDS (Scheme 14 c). Intermediate **13** was found to be the resting state of the catalytic cycle. Protonolysis via H_3_N⋅BH_3_ releases the product, while protonation by free NH_3_ was discarded.

### 3.2 Metal‐Free TH

In the metal‐free reduction reactions with amine–boranes, most of the examples follow concerted TH pathways with the formation of a six‐membered‐ring transition state analogous to the work published by Berke's group on the TH of imines with H_3_N⋅BH_3_,^[14]^ with the exception of a reported stepwise pathway for CO_2_ reduction (vide infra).

The development of pnictogen‐catalyzed TH of azoarenes with H_3_N⋅BH_3_ was initiated by the Radosevich group (Scheme 15 a).^[43]^ The authors reported the synthesis of the T‐shaped strained P^III^ compound **14**, which was able to oxidatively add H_2_ from H_3_N⋅BH_3_ and form the active P^V^ compound **16**. The latter was isolated and proposed to be the active species which enabled this catalytic transformation. However, later computational studies on the reaction mechanism suggested that a P^III^–ligand cooperativity might be in action instead of the initially hypothesized active P^III^/P^V^ redox cycle (Scheme 15 b).^[44]^ Concerted activation of H_3_N⋅BH_3_ with a barrier of Δ*G* ^≠^=27.1 kcal mol^−1^ allowed formation of species **15** (Δ*G*°=13.5 kcal mol^−1^), the active species for the TH of azobenzene to 1,2‐diphenylhydrazine through a concerted six‐membered‐ring TS (Δ*G* ^≠^=28.1 kcal mol^−1^). This step can therefore be denoted as the RDS of the reaction.

Dimerization of **15** resulting in formation of the P^V^–hydride species **16**, originally isolated by Radosevich's group, was found to be energetically feasible (Δ*G* ^≠^=8.9 kcal mol^−1^). However, it was determined that **16** was an off‐cycle species for the TH of azobenzene, and other potential mechanistic pathways, for example, insertion of the N=N bond into P−H bond (43.2 kcal mol^−1^) or reduction of azobenzene through ion‐pair interaction with P−H (29.4 kcal mol^−1^), were discarded because of the high energy requirements.

**Scheme 16:**
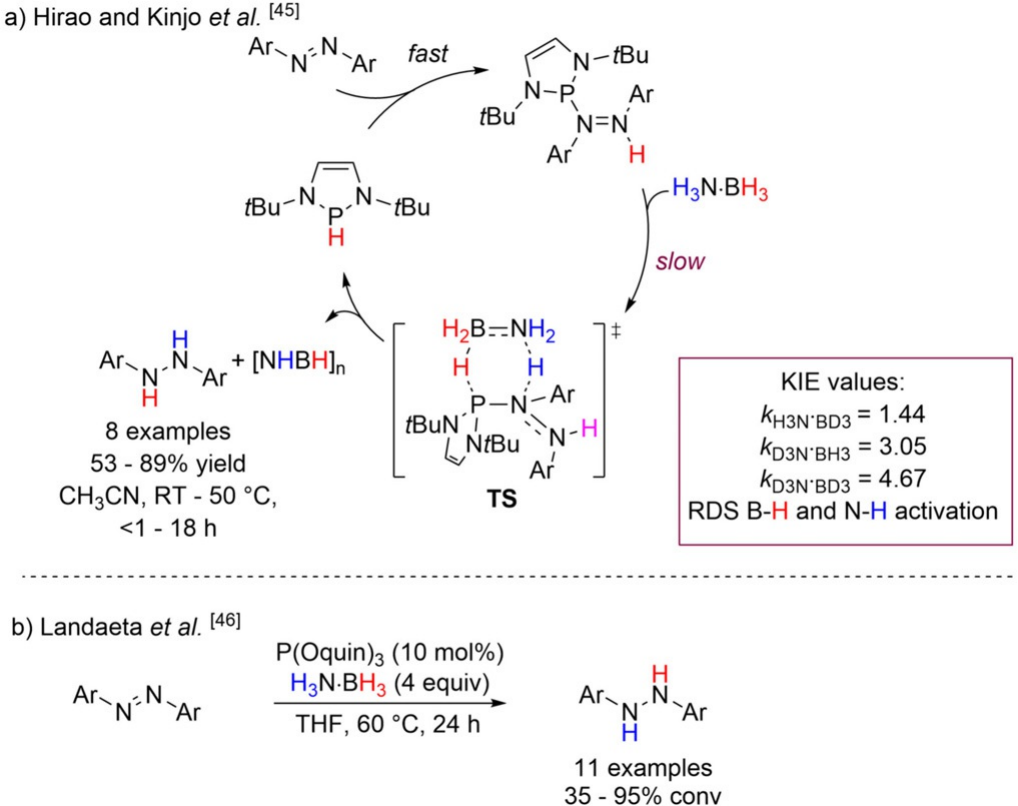
a) Proposed catalytic cycle for the TH of azoarenes by DPAs with H_3_N⋅BH_3_; b) phosphite reduction of azoarenes with H_3_N⋅BH_3_.

Following these findings, Hirao and Kinjo reported the catalytic reduction of azoarenes with H_3_N⋅BH_3_ and 5 mol % 1,3,2‐diazaphospholenes (DAPs) (Scheme 16 a).^[45]^ This process was more efficient than Radosevich's procedure, where catalysis was generally faster. A proposed catalytic cycle was presented which starts with insertion of the P−H bond into N=N, followed by hydrogenolysis of the exocyclic P−N bond via H_3_N⋅BH_3_. The latter hydrogen transfer was found to proceed through a concerted six‐membered‐ring TS with protic and hydridic hydrogen transfer to N and P atoms, respectively. This pathway was energetically feasible (Δ*G* ^≠^=25.2±4.2 kcal mol^−1^, Δ*H* ^≠^=21.8±2.2 kcal mol^−1^, and Δ*S* ^≠^=−11.6±6.8 e.u.), with oligomerization of H_3_N⋅BH_3_ accounting for the slightly endergonic nature of the reaction. Additional stepwise pathways were analyzed but discarded as they were found to be more energetically demanding. DKIE analysis for the reduction of azobenzene to 1,2‐diphenylhydrazine was carried out using isotopically labeled H_3_N⋅BH_3_. These experiments revealed normal KIEs with D_3_N⋅BH_3_ (3.05), H_3_N⋅BD_3_ (1.44), and D_3_N⋅BD_3_ (4.67). These values demonstrated that B–H and N–H activation of H_3_N⋅BH_3_ are involved in the RDS. When H_3_N⋅BD_3_ was used, incorporation of deuterium was found to be selective for 1,3,2‐diazaphospholene recovered at the end of the reaction, with traces of PH_3_ formation. The role of phosphane in the reaction was not investigated further.

Recent findings of Landaeta and co‐workers on a similar reduction reaction with an acyclic phosphite precatalyst (Scheme 16 b)^[46]^ substantiated that the reduction of azoarenes with pnictogenide precatalysts is prone to an associative mechanism. The authors presented a detailed mechanistic study of the latter confirming, through DKIE values following the same trend as that in Hirao and Kinjo's work (*k* ${}_{H{}_{3}N\cdot \mathrm{BD}{}_{3}}$ <*k* ${}_{D{}_{3}N\cdot \mathrm{BH}{}_{3}}$ <*k* ${}_{D{}_{3}N\cdot \mathrm{BH}{}_{3}}$ ), that concerted B−H and N−H bond breaking was involved in the slowest reaction step.^[47]^

**Scheme 17:**
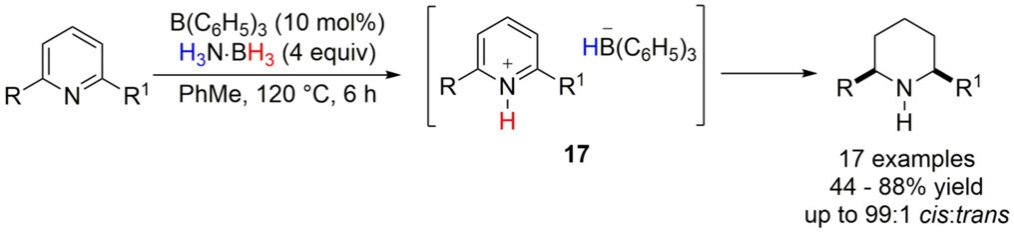
TH of pyridine with ammonia–borane using BCF precatalyst.

With the rise of frustrated Lewis pair (FLP) chemistry and following the finding that H_2_ is released from H_3_N⋅BH_3_ initiated by B(C_6_H_5_)_3_ (BCF),^[48]^ TH reactions were performed using Lewis acid activation. In one of the first examples, Du and co‐workers^[49]^ reported the activation of H_3_N⋅BH_3_ with BCF (10 mol %) to obtain a stereoselective reduction of pyridines to piperidines (Scheme 17). The authors proposed the formation of a zwitterion species of type **17** resulting from hydride abstraction of H_3_N⋅BH_3_ from the Lewis base/Lewis acid adduct. A similar hypothesis was proposed by Shi and co‐workers in the development of *N*‐heteroarene reduction,^[50]^ by Xiao and co‐workers for the deoxygenation of amides and lactams,^[51]^ and by Zhong and co‐workers for the reductive amination of ketones.^[52]^

**Scheme 18:**
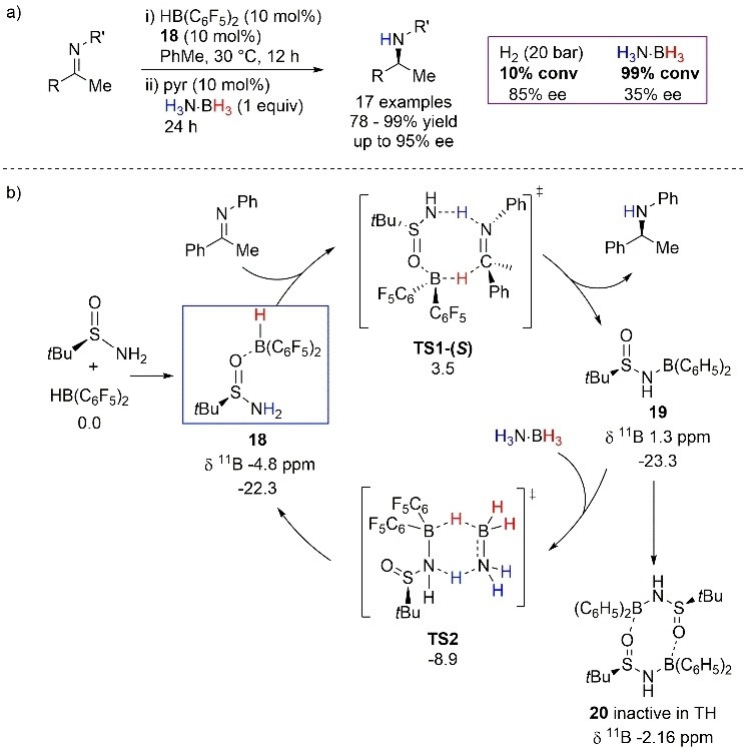
a) Imine TH with FLP pair using H_3_N⋅BH_3_ as a sacrificial reductant; b) detailed mechanistic studies with calculated free Gibbs energies in toluene in parenthesis.

Du, Meng, and co‐workers reported an asymmetric and stereoselective TH of imines using H_3_N⋅BH_3_ (Scheme 18 a).^[53]^ The authors exploited the idea of zwitterion ion pair by using chiral *tert*‐butyl sulfonamide and Piers’ borane HB(C_6_H_5_)_2_, originally finding success in the stoichiometric reaction and then transposing it into a catalytic process (10 mol %). It is worth noting that H_2_ (20 bar) was not an efficient reductant, with low conversion found compared to the reaction performed with H_3_N⋅BH_3_ (10 % versus 99 % conversion after 20 h). The B‐O isomer **18** was proposed to be the active intermediate and ^11^B NMR analysis of the catalytic reaction allowed identification of the species, which showed a broad signal at −4.8 ppm (Scheme 18 b). Formation of this isomer, from addition of Piers’ borane and the sulfonamide, was also found to be the most likely by DFT calculations. From this active species TH occurred via the eight‐membered **TS1‐(*S*)**, which is responsible for the enantioinductive step. Release of chiral amine and formation of dehydrated species **19** were confirmed by NMR spectroscopy, with **19** having a characteristic ^11^B NMR signal at 1.3 ppm. Stoichiometric experiments showed that **19** could quickly regenerate active catalyst **18** in the presence of H_3_N⋅BH_3_ via an energetically viable concerted pathway (Δ*G* ^≠^=14.4 kcal mol^−1^). The authors hypothesized the role of **19** as a Brønsted acid initiator for the reaction, although the barrier found for this process (Δ*G* ^≠^=29.0 kcal mol^−1^) was not comparable to that of the FLP mechanism. Moreover, **20**, a dimer of **19**, was isolated and proven to be unable to perform catalysis and described as an off‐cycle species for the TH of imines.

**Scheme 19:**
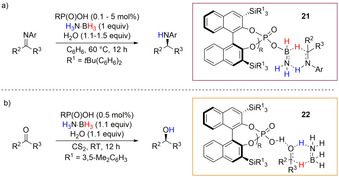
a) Phosphoric acid reduction of imines with H_3_N⋅BH_3_ and proposed enantioselective TS from chiral amine–borane complex **21**; b) phosphoric acid reduction of ketones with H_3_N⋅BH_3_ and proposed enantioselective TS **22**.

Further advancement of the asymmetric TH of imines and ketones with H_3_N⋅BH_3_ was described by Du and co‐workers when they used enantioenriched phosphoric acid (Scheme 19 a,b).^[54]^ The chiral ammonia–borane complex **21** was isolated but, while it was found to be off‐cycle for ketone reduction (Scheme 19 b), it was proven to be an active intermediate in the TH of imines by stoichiometric studies and DFT calculations (Scheme 19 a). Interestingly for both processes, DFT calculations supported the formation of a six‐membered‐ring TS which accounted for substrate activation by amine–borane chiral complex **21** for imine reduction, while it involves substrate activation by phosphoric acid to form species **22**, followed by TH from H_3_N⋅BH_3_ for ketone reduction.

**Scheme 20:**
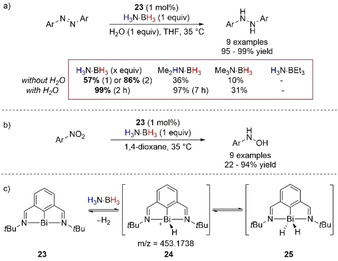
a) Bismuthine‐catalyzed TH of azoarenes and b) nitroarenes with H_3_N⋅BH_3_ and details regarding the effect of H_2_O addition; c) Bi^III^ hydride species **23** and **24** speculated as part of the mechanistic cycle.

A recent development of catalysis by pnictogens was reported by Cornella and co‐workers who used a well‐defined Bi^I^ compound to deliver TH of azo‐ and nitroarenes (Scheme 20 a,b).^[55]^ The authors suggested an elusive bismuthine(III) hydride species might form by oxidative addition to Bi^I^ (Scheme 20 c); the evidence for such species was given by analyzing the catalytic reaction by high‐resolution mass spectrometry (HRMS), which showed an adduct at 453.1738 g mol^−1^ assigned to a cationic Bi^III^ monohydride complex **24**. Release of H_2_ was evident when the dehydrogenation of H_3_N⋅BH_3_ was performed with bismuthine species. However, no further description of the role of H_2_ in the reaction was made, leaving an open question whether the reduction performed is classical TH or hydrogenation. The role of H_3_N⋅BH_3_ was found to be crucial in the reaction with 57 % conversion found after 16 h, while switching to amine–boranes, for example, Me_3_N⋅BH_3_ or H_3_N⋅BEt_3_, resulted in lower conversion (10 %) or no conversion after the same reaction time. H_2_O played an important, if undefined, role in the TH of azoarenes, and 1 equiv was added to the reaction mixture in the reduction of azoarenes to decrease the reaction time (from 16 to 2 h) and the amount of reductant (1 equiv instead of 2). The combination of a stoichiometric amount of H_2_O and H_3_N⋅BH_3_ increased conversion to 99 % after 2 h from 57 % when only 1 equiv of reductant was used and to 86 % after 16 h when 2 equiv of H_3_N⋅BH_3_ was used. Reasonably, the authors could not further discriminate the role of H_2_O through isotope labeling experiments, because of the potential fast exchange with H_3_N⋅BH_3_. However, DKIE analysis obtained studying the initial conversion of azobenzene into 1,2‐diphenylhydrazine showed a large primary kinetic isotope effect, with *k* ${}_{D{}_{3}N\cdot \mathrm{BH}{}_{3}}$ =1.63, *k* ${}_{H{}_{3}N\cdot \mathrm{BD}{}_{3}}$ =3.94 and *k* ${}_{D{}_{3}N\cdot \mathrm{BD}{}_{3}}$ =7.05 which indicated a concerted TS as RDS, reminiscent of the results found for DPAs and phosphite‐catalyzed TH (Scheme 16).

**Scheme 21:**
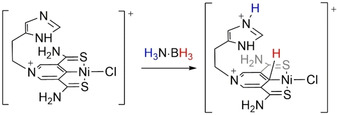
Scorpionate SCS–Ni pincer complexes and the proposed activation of H_3_N⋅BH_3_.

A computational exploration of the potential of SCS‐Ni pincer complexes in the TH of acetone, acetophenone, and methanamine with H_3_N⋅BH_3_ has also been reported.^[56]^ The calculations reveal that a proton‐coupled hydride transfer is the more energetically demanding step for the reduction of ketones, while a stepwise hydride and proton transfer might occur in the TH of imines (Scheme 21). The key to this transformation was the imidazolium substituent on the SCS ligand that acted as proton shuttle. The reactivity of these complexes was compared to the reactivity of lactate racemate,^[57]^ where the potential role of the metal center might be solely to stabilize the molecular entity which needs to perform the transformation, as observed by others.^[58]^ Future experimental and mechanistic details would be of great interest to clarify and test the potential of this theoretical exploration.

**Scheme 22:**
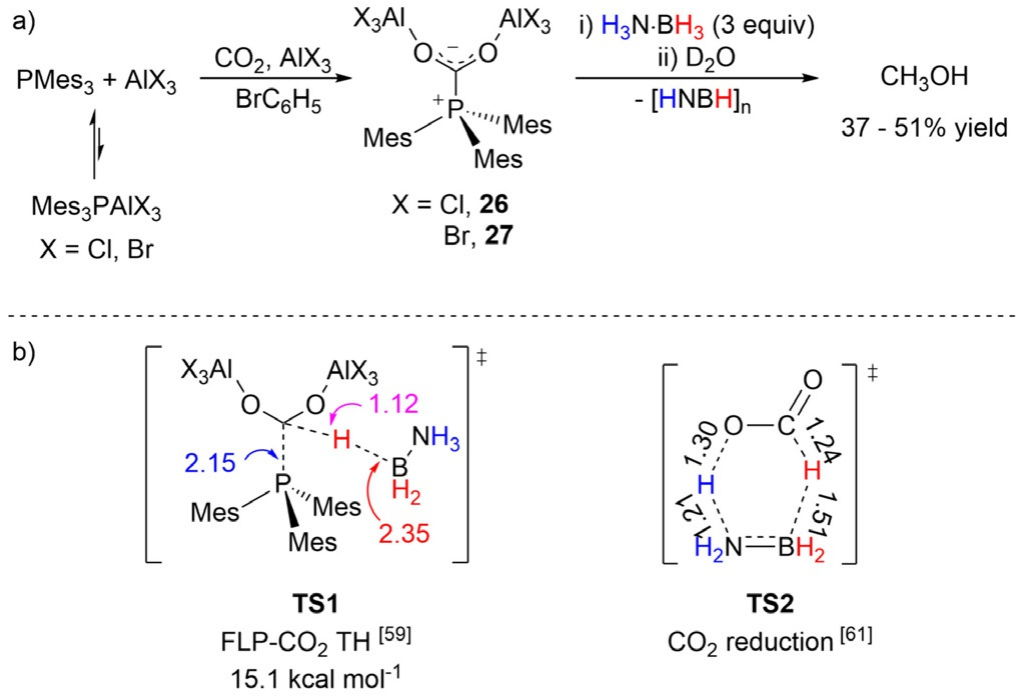
a) CO_2_ reduction by FLP and b) rate‐determining transition states of the catalyzed and uncatalyzed reactions with calculated bond lengths expressed in Å.

The final example of TH is that of CO_2_ and it differs slightly from the non‐metal‐catalyzed TH examples reported so far. Initially reported by Ménard and Stephan in 2010 (Scheme 22 a),^[59]^ the stoichiometric reduction of CO_2_ to MeOH was performed by tris(2,4,6‐trimethylphenyl)phosphine (PMes_3_) and an excess of AlX_3_ (X=Cl, Br) FLP species; the formation of FLP–CO_2_ adducts **26** and **27** was confirmed by NMR spectroscopy and X‐ray analysis, and they could be further reduced with H_3_N⋅BH_3_ and quenched with H_2_O. The reduction reaction was found to be facile allowing a moderate yield (37–51 %) of MeOH after 15 min at room temperature. The mechanism of this TH was further studied by computational methods by Paul and co‐workers (Scheme 22 b).^[60]^ The reduction of CO_2_ to liquid fuel was found to be initiated by interaction of the hydridic B‐H with the C1 atom of FLP–CO_2_ and PMes_3_ displacement. The energy barrier to **TS1** of 15.1 kcal mol^−1^ was in line with the mild experimental conditions. Subsequent reduction steps were calculated to be driven by B–H activation, with the exception of the hydrolysis step which allows final C−O bond cleavage, which was postulated to be facilitated by H_3_N⋅BH_3_ or dehydrated oligomers, as found by Webster and co‐workers (Scheme 13, Section 3.1). Interestingly, the authors compared this mechanism to the uncatalyzed reduction of CO_2_ to formic acid,^[61]^ for which they could locate a six‐membered‐ring TS, which favors the hypothesis of a concerted mechanism.

**Scheme 23:**
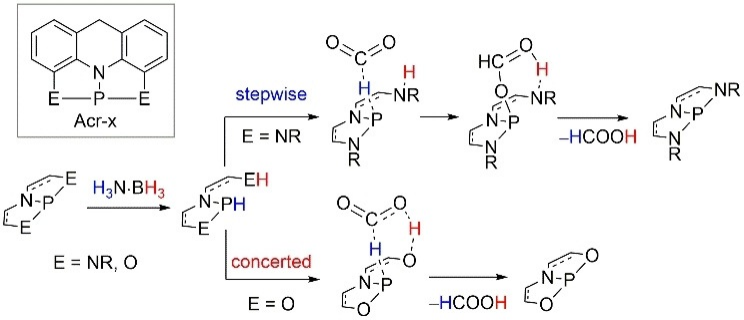
Simplified reaction mechanism for the reduction of CO_2_ with H_3_N⋅BH_3_ by pincer‐P^III^ complexes.

In a creative theoretical experiment, Maeda, Sakaki, and co‐workers described the use of computationally designed ONO and NNN pincer P^III^ compounds for the activation of CO_2_ (Scheme 23).^[62]^ The author's calculations were found to be in line with literature findings, with ligand‐P^III^ dehydration of H_3_N⋅BH_3_ being the RDS (19.7 kcal mol^−1^), followed by CO_2_ reduction. The latter proceeded via a concerted pathway when optimizations were performed with ONO‐P pincer ligands. When calculations were focused on NNN‐P pincer ligands, a stepwise coordination of formate to P‐H followed by reduction to formic acid was most likely to be in action.

### 3.3 Supramolecular and Heterogeneous Examples

Reaction mechanisms for TH reactions with supramolecular and heterogeneous systems are somewhat less studied and the lack of mechanistic investigation does not allow discrimination between these systems as being either TH or standard hydrogenation reactions. Therefore, we will highlight in this section only the reactions where the mechanism has been proven to be a classical TH by amine–boranes.

Initial reports of supramolecular systems of amine–boranes to perform reduction reactions were published in 1984.^[63]^ Allwood and co‐workers explored the formation of a supramolecular adduct formed between substituted chiral 18‐crown‐6‐ethers and H_3_N⋅BH_3_; the adducts were isolated and characterized by X‐ray diffraction, and were found to be active in the enantioselective reduction of ketones with selectivities up to 67 % *ee*.

**Scheme 24:**
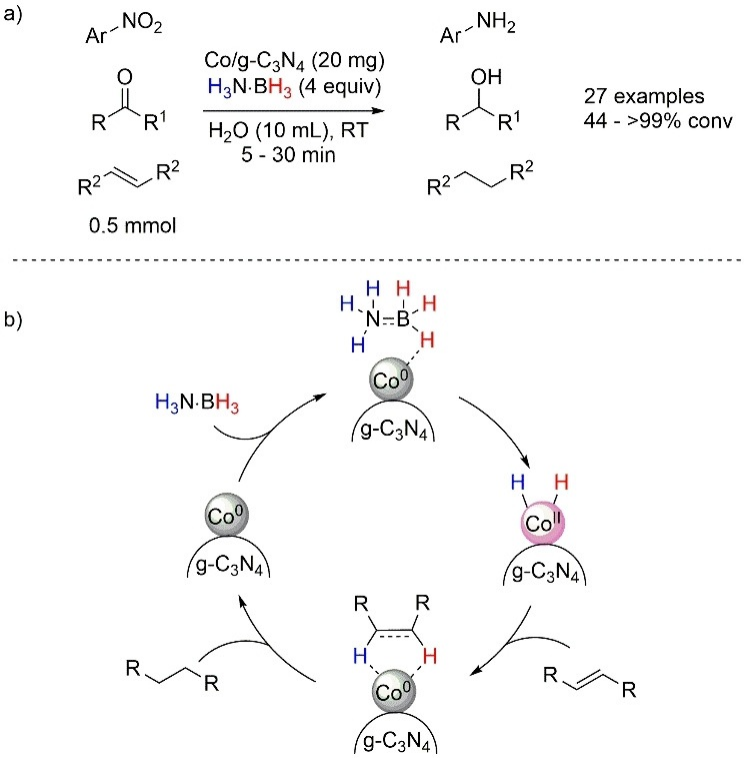
a) Co/g‐C_3_N_4_‐catalyzed TH of nitro compounds, arenes, and ketones; b) postulated catalytic cycle.

A rare example of heterogeneous TH was reported by Li and co‐workers in 2015 (Scheme 24 a).^[64]^ The authors built cobalt nanoparticles on graphitic carbon nitride dyad (Co/CN or Co/g‐C_3_N_4_) on a mesoporous carbon nitride as the catalyst support which resulted in a hybrid structure of amorphous shells (Co^2+^) and metallic core (Co^0^) as confirmed by X‐ray photoelectron spectroscopy (XPS). The Co/CN material was highly active in the TH of nitroarenes, olefins, ketones, and aldehydes with H_3_N⋅BH_3_ as the hydrogen transfer agent. The TH was efficiently performed at room temperature in less than 1 h. The reaction supported small‐scale application with 20 mg of catalyst per 0.5 mmol of substrate, but could also be scaled up by a factor of 10. It is important to note that when the reaction was performed in an atmosphere of H_2_ (1 bar), there was no conversion after 12 h. A potential Co^2+^/Co^0^ redox pair was postulated to be involved in the reaction mechanism (Scheme 24 b); however, the formation of CoH_*x*_ and/or Co–amidoborane intermediates could not be excluded.

**Scheme 25:**
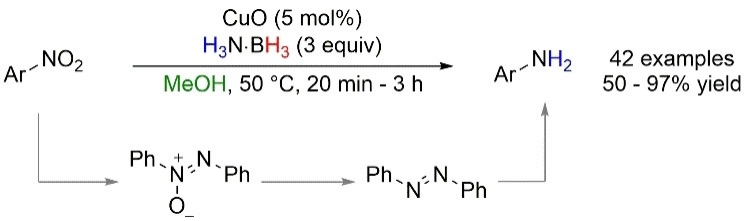
CuO‐catalyzed TH of nitroarenes.

Non‐supported commercially available CuO was used for the TH of nitro compounds (Scheme 25).^[65]^ H_3_N⋅BH_3_ was the only reducing agent capable of performing the reaction, while NaBH_4_, hydrazine, acetic acid, and H_2_ showed limited conversion (<10 %). The reaction was run in alcoholic solvents, with MeOH being optimum; when CD_3_OD was used, no deuterium incorporation was found in the final product, which discounted the solvolysis of H_3_N⋅BH_3_ in the reaction mixture. Reduced intermediates, such as azoxybenzene and diazobenzene, which were found by ^1^H NMR analysis, favored a stepwise TH mechanism.

**Scheme 26:**
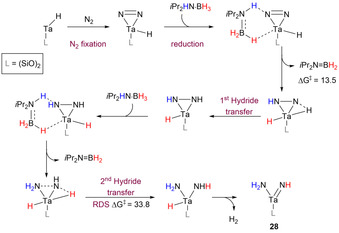
Details of the N_2_ splitting at Ta supported on silica with *i*Pr_2_HN⋅BH_3_ reducing agent; free energies are given in kcal mol^−1^.

In a detailed computational exploration, Ankan and co‐workers described the potential fixation of N_2_ onto tantalum atoms supported on a silica surface, and its further reduction using *i*Pr_2_HN⋅BH_3_ (Scheme 26).^[66]^ The authors analyzed the reduction in the presence of *i*Pr_2_HN⋅BH_3_ as the reducing agent, and noticed that the latter, in contrast to H_3_N⋅BH_3_, does not oligomerize once dehydrogenated; this could allow the aminoborane product *i*Pr_2_N=BH_2_ to be rehydrogenated to the amine–borane equivalent. Concerning the fixation/reduction of nitrogen on tantalum, the author proposed the formation of a tantalum amido imido intermediate [(≡SiO)_2_Ta(=NH)(NH_2_)] (**28**) as the end‐product of the reaction, as found by others when analyzing the fate of N_2_ reduction with molecular H_2_.^[67]^ N_2_ could approach the supported Ta atoms and be activated through a relatively low activation barrier of 14.5 kcal mol^−1^, resulting in elongation of the N−N bond length (1.20 Å) compared to free N_2_ (1.09 Å), indicating activation. Further stepwise proton and hydride transfer to the N−Ta bond allowed formation of a diazenido species, which was further reduced to [(≡SiO)_2_TaH(NHNH)] by hydride migration from Ta to N. A second equivalent of amine–borane could further activate species **28** and form [(≡SiO)_2_TaH_2_(NH_2_NH)]; the following second hydride migration from the Ta centre was predicted to be RDS with an activation energy Δ*G* ^≠^=33.8 kcal mol^−1^ to form [(≡SiO)_2_Ta(=NH)(NH_2_)] **28**. This event was found to be energetically favored at 13.5 kcal mol^−1^ compared to other models using molecular H_2_ as reductant with an activation barrier of 43 kcal mol^−1^.^[68]^ Furthermore, the author predicted that the reaction could be implemented experimentally at low temperature (160–170 °C) and suggested a way to circumvent catalyst decomposition via exposing the surface to N_2_ at high pressure in order to maximize fixation and subsequently allow more facile hydrogenation to occur. However, even though the results looked promising, no further experimental evidence to disprove the authors’ findings have been reported yet; this is certainly an encouragement to expand on the topic.

Recently Jiang and co‐workers synthesized a core–shell CuPd@ZIF‐8 composite, with a cubic CuPd core and a MOF shell, and applied the system to the selective semi‐TH of alkynes with H_3_N⋅BH_3_.^[69]^ This system is notable because of the synergistic behavior of the Cu and Pd centres, which allows selective absorption of H_3_N⋅BH_3_ (−1.38 eV on Cu versus −1.49 eV on Pd) and phenylacetylene (−2.43 on Pd versus −0.61 eV on Cu), respectively. The MOF shell protects the core, decreasing potential chemical etching of Cu nanocubes, even after five consecutive runs. Deuterium labeling experiments allowed assessment of the role of H_3_N⋅BH_3_ in the system; a first order rate dependence on the reductant was observed under catalytic conditions for the reduction of phenylacetylene, with a KIE of 4.08 using H_3_N⋅BD_3_, indicating that the B–H activation is rate determining. Incorporation of deuterium occurs also in the presence of D_3_N⋅BH_3_, while solvolysis was not in action with no deuterium incorporation into styrene when MeOD was used instead. The negligible capacity of H_2_ to perform the reduction was also described and further DFT calculations on the catalytic system could define clearly that a classical TH reaction is in effect.

## 4 Solvolysis of Amine–Boranes in Nonclassical TH Reactions

In this section, we are concerned with the parallel/alternative route that can occur once H_3_N⋅BH_3_ dissociates into free NH_3_ and the solvent adduct of BH_3_—that is the reduction of an unsaturated bond by an initial hydroboration step followed by protic solvent work‐up. This route should be categorized as nonclassical TH as the protic hydrogen is donated from the solvent and not the amine counterpart, but importantly, nor are the hydrogens due to H_2_ released from the solvolysis of H_3_N⋅BH_3_.^[15]^ Similar to Section 3, the literature reviewed here, opens up some ambiguity into the precise mechanism operating. We have inferred a solvolysis pathway occurring where the authors themselves have not classified whether classical or nonclassical TH is undergoing in their systems.

Early examples of solvolysis of amine–boranes in reduction reactions have been reported by Jones using Me_3_N⋅BH_3_ to reduce 4‐*tert*‐butylcyclohexanone in benzene followed by aqueous work up.^[70]^ In 1971, Borsch and Levitan investigated the asymmetric reduction of ketones with optically active phenethylamine–borane with high conversion but very poor optical purity of the final product.^[71]^ These early examples, although they did not provide a great deal of mechanistic insight, showed the potential of amine–boranes as reducing agents in combination with aqueous work‐up or action of solvolysis.

### 4.1 Uncatalyzed Solvolysis

**Scheme 27:**
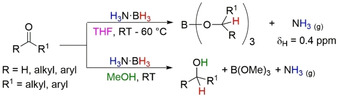
Solvolysis of H_3_N⋅BH_3_ to effect hydroboration of aldehydes and ketones in THF (top) and hydrogenation of aldehydes and ketones in MeOH (bottom).

Continuing on their previous work of uncatalyzed TH of polarized bonds (Section 2), and on metal catalyzed TH of olefin (Section 3.1) Berke and co‐workers explored the reactivity of aldehydes and ketones with H_3_N⋅BH_3_ (Scheme 27).^[72]^ Rather than observing analogous TH reactions in THF they found only hydroboration of the C=O moiety to form borate esters. The presence of free NH_3_ was also observed in situ by NMR spectroscopy (δ_H_=0.4 ppm), suggesting an alternative mechanism was operating in contrast to those previously reported in Section 2. When benzophenone was used as the model substrate, deuterium labeling experiments showed only deuterium incorporation at the carbon position of the C=O unit when H_3_N⋅BD_3_ or D_3_N⋅BD_3_ was used, confirming the “spectator” role of NH_3_ (e.g. D_3_N⋅BH_3_ led to no deuterium incorporation into the product). Furthermore, similar reactivities were observed with H_3_B⋅THF as the hydrogen source. DKIE experiments showed normal DKIEs (*k* ${}_{D{}_{3}N\cdot \mathrm{BH}{}_{3}}$ /*k* ${}_{H{}_{3}N\cdot \mathrm{BH}{}_{3}}$ =1.74 and *k* ${}_{D{}_{3}N\cdot \mathrm{BH}{}_{3}}$ /*k* ${}_{D{}_{3}N\cdot \mathrm{BD}{}_{3}}$ =1.10) with D_3_N⋅BH_3_ and normal DKIEs (*k* ${}_{H{}_{3}N\cdot \mathrm{BD}{}_{3}}$ /*k* ${}_{H{}_{3}N\cdot \mathrm{BH}{}_{3}}$ =1.28 and *k* ${}_{H{}_{3}N\cdot \mathrm{BD}{}_{3}}$ /*k* ${}_{D{}_{3}N\cdot \mathrm{BD}{}_{3}}$ =1.49) with H_3_N⋅BD_3_. These experiments suggest the dissociation of H_3_N⋅BH_3_ is the RDS and the values are indicative of a secondary KIE due to changing geometry at the N and B atoms. The H_3_B⋅THF species can then undergo standard hydroboration reactions with aldehydes and ketones to form borate esters.

Performing the reaction in MeOH, Berke found formation of the desired primary and secondary alcohols along with B(OMe)_3_ and free NH_3_ as the by‐products. It was postulated that dissociation of H_3_N⋅BH_3_ would be the RDS to form free NH_3_ as a spectator molecule and BH_3_ as the reagent. BH_3_ could immediately form an adduct with the C=O moiety of the substrate and hydroboration would form the borate ester intermediate which then undergoes methanolysis to give the products. Alternatively, a MeOH⋅BH_3_ adduct could form after dissociation and undergo direct hydrogenation via a double H transfer with the protic hydrogen coming from the alcohol.^[28]^ Deuterium labeling experiments were unable to distinguish between these two pathways. However, using MeOD confirmed that the deuterium incorporation at the O atom of the carbonyl moiety was solely from the solvent and again indicating that this reaction *does not* undergo a classical TH process.

**Scheme 28:**
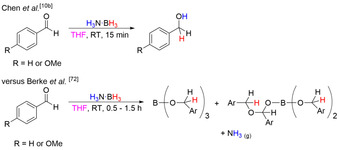
Products reported by Chen and co‐workers^[10b]^ versus those reported by Berke and co‐workers^[72]^ for the reduction of benzaldehyde and 4‐methoxybenzaldehyde in THF with H_3_N⋅BH_3_.

It is worth noting that in countering studies, Chen and co‐workers found the formation of the primary alcohols when reacting H_3_N⋅BH_3_ with a number of aromatic aldehydes in THF (Scheme 28),^[10b]^ and *not* formation of the borate ester. Direct comparisons with Berke's work,^[72]^ where both studies used the same substrates (benzaldehyde and 4‐methoxybenzaldehyde) under same conditions, revealed the different results from the two groups. Following the reaction by multinuclear NMR and FTIR spectroscopy, Chen found no evidence of NH_3_ formation and deuterium labeling experiments confirmed participation of both the protic and hydridic hydrogens from H_3_N⋅BH_3_.

Considering the divergent reactivities displayed in these two investigations in reactions of H_3_N⋅BH_3_ with aldehydes in THF, it would be pertinent to probe the energetic difference between the hydroboration pathway and the classical TH pathway. Additionally, the energetic difference between dissociation of H_3_N⋅BH_3_ in THF compared to that in MeOH would also provide greater insight into solvent effects. It is worth recalling that Berke and co‐workers experimentally showed that no deuterium scrambling occurred when H_3_N⋅BH_3_ was heated with D_3_N⋅BD_3_ at 60 °C for several hours or at room temperature for several days in THF, suggesting a high barrier for dissociation.^[14]^ In 2020, Zhang, Ma, and co‐workers published a DFT study on the reduction of benzaldehyde with H_3_N⋅BH_3_.^[73]^ The authors first examined S_N_1‐ versus S_N_2‐type processes for the dissociation of H_3_N⋅BH_3_, for a reaction involving THF, MeOH, and benzaldehyde. The S_N_1 pathway was the most favorable route to the common adduct, PhCHO⋅BH_3_, with minor differences in the energies in THF (Δ*G* ^≠^=23.5 kcal mol^−1^) and MeOH (Δ*G* ^≠^=24.5 kcal mol^−1^). Furthermore, the RDS in all the S_N_2 routes was after the initial H_3_N⋅BH_3_ dissociation and involved a second dissociation of the BH_3_⋅solvent adduct to form PhCHO⋅BH_3_. Therefore, only the boron counterpart, and not NH_3_, appears to be involved, which would contradict the observed normal DKIE effects with D_3_N⋅BH_3_ or D_3_N⋅BD_3_ reported by Berke and co‐workers.^[72]^

**Scheme 29:**
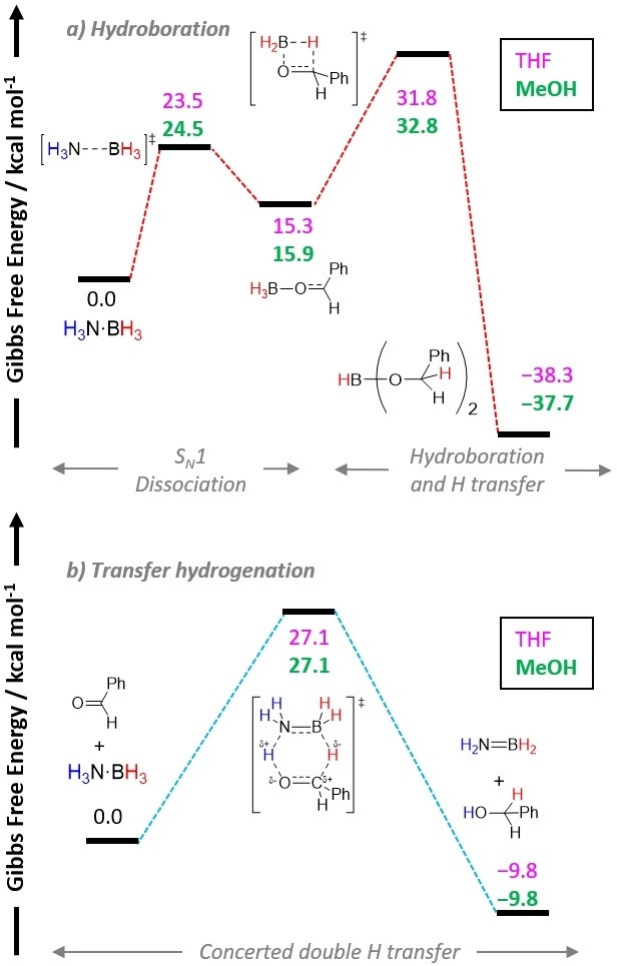
Simplified reaction profile of a) hydroboration of benzaldehyde and b) TH of benzaldehyde with H_3_N⋅BH_3_.

**Scheme 30:**
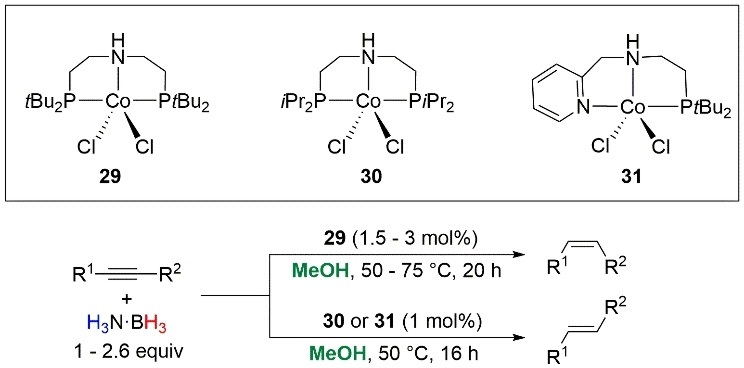
Stereo‐ and chemoselective hydrogenation of alkenes mediated by PNP and NNP Co pincer complexes.

From the PhCHO⋅BH_3_ species, a hydroboration step (THF, Δ*G* ^≠^=31.8 kcal mol^−1^; MeOH, Δ*G* ^≠^=32.8 kcal mol^−1^) was found representing the RDS of the pathway (Scheme 30 a). In comparison, the direct TH route from H_3_N⋅BH_3_ was found to be more kinetically favorable, with the RDS involving the concerted double H transfer (Δ*G* ^≠^=27.1 kcal mol^−1^) (Scheme 29 b). The small difference of 4.7 kcal mol^−1^ between the TH route and hydroboration may suggest that there is some interchangeability between the two pathways depending on the product‐determining step, and potentially could explain the difference in reactivity observed by Berke's group and Chen's group for different aldehydes used in their investigations (Scheme 29).

### 4.2 Homogeneous Mediated Solvolysis

In 2016 Liu, Luo, and co‐workers published the TH of alkynes to *cis*‐ and *trans*‐alkenes selectively using PNP‐ and NNP‐type Co‐pincer complexes.^[39]^ Controlling the steric profile around the cobalt center by altering the groups on the pincer ligands allowed them to access good chemo‐ and stereoselective transformation of numerous alkenes (Scheme 30). The role of H_3_N⋅BH_3_ was seemingly just as the borohydride source. Control and optimization reactions confirmed: 1) A Co catalyst was necessary for the conversion of the alkyne; 2) The reaction was most likely homogeneous under Hg poisoning testing; 3) It was important to use H_3_N⋅BH_3_ as the boron source rather than other conventional borohydrides (NaBHEt_3_, NaBH_3_CN, Me_2_S⋅BH_3_, NaBH(OAc)_3_, Me_2_HN⋅BH_3_); 4) The reaction in alcohols had the higher activity than that in THF or toluene, with MeOH chosen as the preferred solvent.

In order to identify the hydrogen source, deuterium labeling experiments were performed. When diphenylacetylene was used as the model substrate, CD_3_OH showed no deuterium incorporation into the product, but CD_3_OD allowed the isolation of the monodeuterated *trans*‐1,2‐diphenylethene. The reaction of H_3_N⋅BH_3_ with 1 mol % **31** under standard conditions with and without 1 equiv of diphenylacetylene always gave B(OMe)_3_ as the product with formation of H_2_ observed. Without any catalyst or substrate, formation of B(OMe)_3_ in only 5 % yield was observed after 16 h at 50 °C, suggesting the methanolysis of H_3_N⋅BH_3_ is a catalytic process in this system. The group also demonstrated that without H_3_N⋅BH_3_ no product was observed, so MeOH alone could not act as the hydrogen source.

**Scheme 31:**
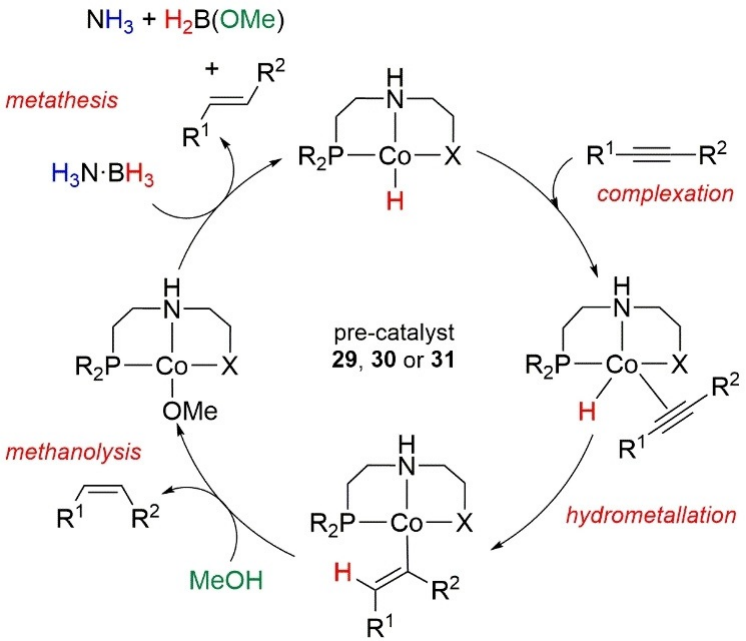
Postulated reaction mechanism of the Co‐catalyzed semihydrogenation of alkynes.

A plausible mechanism was proposed (Scheme 31) based on all the experimental evidence, suggesting the role of H_3_N⋅BH_3_ was to generate the active [Co–H] species, which hydrometalates the alkyne across the triple bond to generate an alkenyl cobalt complex. Methanolysis of the Co−C bond releases the *cis*‐alkene product and forms a [Co–OMe] complex, observed by NMR spectroscopy. Regeneration of the active [Co–H] species is enabled by H_3_N⋅BH_3_ and after 3 turnovers can give B(OMe)_3_ as the by‐product. The authors also propose the competitive isomerization cycle to give the *trans*‐alkene product from the common [Co–H] species. It is worth noting that the use of NaBH_4_ also gave successful results in the optimization reaction and would further corroborate the spectator role of the amine counterpart in H_3_N⋅BH_3_. However, the lack of success when using NaHBEt_3_, a stronger hydride donor, to generate the [Co–H] species is somewhat surprising given the precedence^[74]^ and may allude to a more complex process or alternative process operating to form the active [Co–H] species. Using D_3_N⋅BD_3_ may help to confirm the formation of a [Co–D] species, adding more weight to the mechanism. Furthermore, a control experiment under an atmosphere of H_2_ would be informative and allow further scrutiny whether hydrogenation from H_2_ participates in the mechanism.

The solvolysis of H_3_N⋅BH_3_ to effect reduction reviewed in this section can therefore be viewed as nonclassical TH reactions. The role of the H_3_N⋅BH_3_ is akin to that of borohydride reagents to reduce and activate the catalyst and to regenerate the active metal hydride complex during the cycle, with the NH_3_ component not partaking in the active cycle. Instead, proton transfer is from the protic solvent, namely MeOH, and formation of B(OMe)_3_ or H_3_N⋅B(OMe)_3_ is observed as the by‐product. Deuterium labeling experiments, using alternative borohydride sources and using tertiary ammonia boranes (R_3_N⋅BH_3_, R ≠ H), are simple methods in the chemist's toolbox that could be used to determine whether classical TH is taking place or whether hydride transfer and solvolysis is occurring instead.

What is interesting and less understood is the mechanism of activation of the precatalyst by H_3_N⋅BH_3_. These transition metal hydride species are often invoked based on the precedence of related hydrogenation reactions but not further scrutinized within these systems. Parallel DKIE experiments, kinetic experiments, and initial rates would have provided additional invaluable data towards understanding this preactivation step. Inference from transition metal mediated dehydrogenation/dehydrocoupling of H_3_N⋅BH_3_ may be pertinent in this instance.[^12e^, ^12f^, ^15c^, ^75^]

### 4.3 Heterogeneous Mediated Solvolysis

**Scheme 32:**
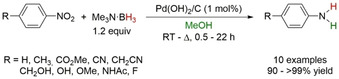
Reduction of nitroaryls with Me_3_⋅BH_3_ mediated by Pd(OH)_2_/C.

The literature around the reduction of unsaturated substrates by methanolysis of amine–boranes under heterogenous conditions is scarce. This may be due in part to the dearth of mechanistic data available in order to determine whether the reactions are simply dehydrogenation of amine–boranes with molecular H_2_ transferred to a surface to participate in subsequent hydrogenolysis. However, in 2001 Couturier and co‐workers reported the methanolysis of primary, secondary, tertiary, and aromatic amine–boranes with Pd/C and Raney Ni at room temperature in MeOH.^[76]^ The absence of protic hydrogens on the tertiary and aromatic amines would confirm that the hydrogen release is due to methanolysis of the amine borane. In follow‐up studies they envisioned the reduction of nitroaryls using amine—boranes, provided the rate of reduction was faster than the rate of H_2_ release.^[77]^ Me_3_N⋅BH_3_ was chosen in this study. Reaction times for the reduction of nitroaryls varied from 0.7–22 h for room temperature reactions (Scheme 32). A control reaction with Me_3_N⋅BH_3_ and 10 mol % Pd(OH)_2_/C showed a reaction time of 20 h for complete methanolysis, which was determined by monitoring the amount of H_2_ released. This provided good evidence that reduction was occurring faster than H_2_ release.

**Scheme 33:**
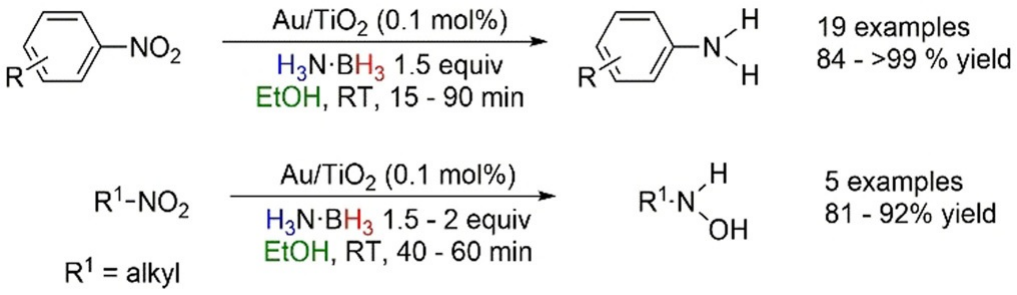
Reduction of nitroarenes and nitroalkanes with H_3_N⋅BH_3_ mediated by Au NPs supported on TiO_2_.

In 2013, Stratakis and co‐workers reported the reduction of nitroarenes and nitroalkanes into anilines and alkylhydroxylamines, respectively, using H_3_N⋅BH_3_ as the reductant and Au NPs supported on TiO_2_ as the catalyst (Scheme 33).^[78]^ Optimization reactions using *p*‐nitrotoluene showed the reaction performed best in EtOH and H_2_O as the solvent with less than 5 % conversion observed with polar aprotic and nonpolar solvents. A control reaction without any Au NPs in EtOH showed no conversion to product. Stratakis et al. noted that the reaction was unlikely to involve H_2_ gas as in related studies by Corma and co‐workers, where high temperatures (100–140 °C) and high pressures of H_2_ (9–25 bar) were required to mediate the chemoselective reduction of nitroarenes by the same catalyst system.^[79]^ Instead, the authors suggested involvement of Au–H species without further scrutiny of the mechanism and they were unable to identify the fate of H_3_N⋅BH_3_ after the reaction. However, based on the solvent optimization reactions, the effect of EtOH indicates that solvolysis pathway might be in operation, but without further experimental evidence, this cannot be substantiated.

**Scheme 34:**
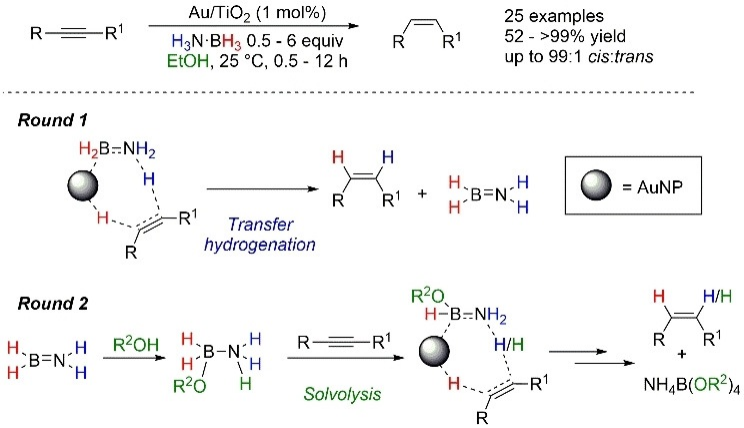
Reduction of alkynes mediated by Au NPs supported by TiO_2_ through classical TH and solvolysis.

Following up on this study, Stratakis and co‐workers expanded the scope of their reaction to report the stereoselective *cis*‐semihydrogenation of alkynes to alkenes (Scheme 34).^[80]^ Solvent optimization of their system found again that aprotic polar solvents and nonpolar solvents resulted in poor conversion, with EtOH again showing the best results. Interestingly adding 5 % v/v H_2_O in THF improved reduction from 11 to >99 % conversion when compared to just using THF. Investigating the reductant source, they found using H_3_N⋅BH_3,_ Me_2_HN⋅BH_3_, and MeH_2_N⋅BH_3_ gave full conversion, but *t*BuH_2_N⋅BH_3_ and Me_3_N⋅BH_3_ resulted in 15 % and no conversion, respectively. Furthermore, using H_3_B⋅SMe_2_ or HBpin also gave no product, which suggested the amine counterpart is important but also both the B–H and N–H are involved with the reduction. Reaction of 0.5 equiv of H_3_N⋅BH_3_ with deuterium‐labeled *p*‐methoxyphenylacetylene to afford the stereoselective *cis*‐addition product indicated a potential concerted addition of the hydrogens. In further studies of the reaction mechanism, ^11^B NMR spectroscopy provided information on the destination of the H_3_N⋅BH_3_ after the reaction. Analysis of the liquid phase using CD_3_OD showed a peak at δ_B_=9.0 ppm, which was assigned as NH_4_B(OCD_3_)_4_; this was the only additional peak observed in the ^11^B NMR spectra at the end of the reaction. Based on their experimental data, they proposed involvement of Au–H species generated from insertion of the B−H bond from H_3_N⋅BH_3_. Importantly, the first double H transfer to the triple bond would therefore arise from N–H and Au–H moieties to release H_2_N=BH_2_ as the by‐product. This would explain the inadequacy of using Me_3_N⋅BH_3_, H_3_B⋅SMe_2_, and HBpin in the reaction. However, H_2_N=BH_2_ can quickly react with the protic solvent (ROH) to form the ammonia alkoxyborane complex, (RO)H_2_B⋅NH_3_, which is anticipated to be more reactive than the parent H_3_N⋅BH_3_. This complex can then undergo an additional round of reduction, with the double H transfer originating from the borane moiety of the complex and proton from the solvent, to finally give the borate salt NH_4_B(OR)_4_ as the by‐product in the reaction. When the reaction was performed using CD_3_OD or THF/D_2_O and *p*‐methoxyphenylacetylene as the substrate, there was 60–65 % deuterium incorporation on both carbon atoms of the styrene moiety, corroborating with the proposed mechanism. This study represents involvement of *both* classical TH and nonclassical TH (solvolysis) processes at different stages of the reaction with the choice of starting amine–borane salient to the success of the reduction reactions. This further highlights the difficulty distinguishing the “true” mechanism in operation of these reactions with amine–boranes as the reductant, as the easy interchangeability of pathways that can be undertaken by the amine–borane based on reaction conditions can cloud the mechanism.

**Scheme 35:**
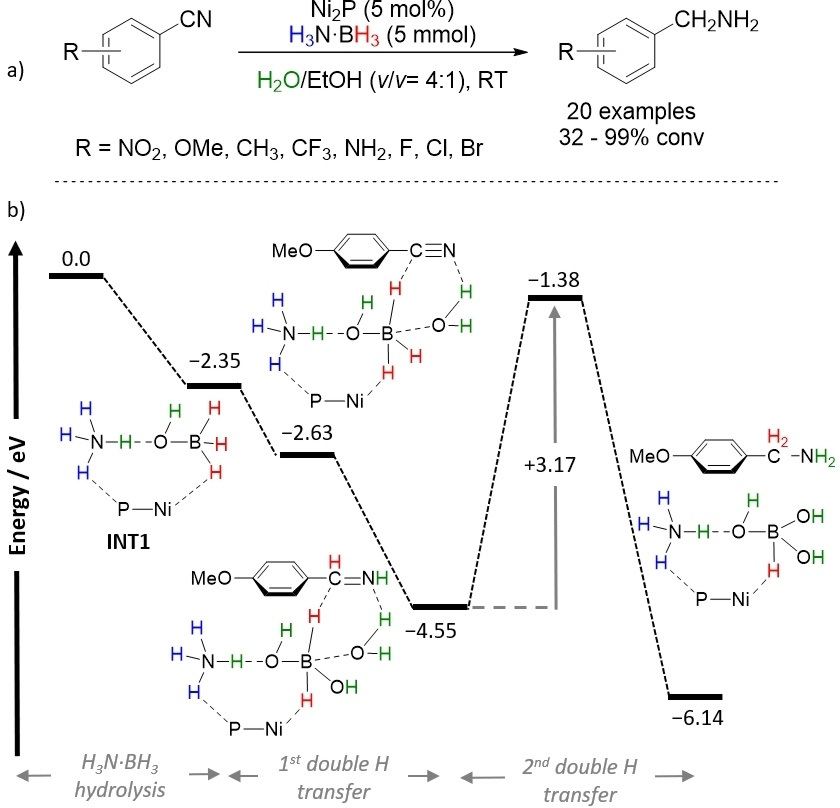
a) Reduction of nitrile mediated by Ni_2_P by H_3_N⋅BH_3_ in EtOH/H_2_O solvent; b) simplified reaction profile of reaction.

In 2017 Fu and co‐workers reported the reduction of nitrile and nitro groups to primary amines using Ni_2_P NPs with H_3_N⋅BH_3_ in a mixed ethanol/water solvent system (1/4*, v/v)* (Scheme 35 a).^[81]^ Control reactions using H_2_ (1 atm) as the hydrogen source showed no formation of product, suggesting that the dehydrogenation of H_3_N⋅BH_3_ is not operating in this system. The reaction mechanism was further probed by DFT calculations using 4‐methoxybenzonitrile as the model substrate with Ni_2_P NPs as the catalyst in water (Scheme 35 b). The initial hydrolysis of H_3_N⋅BH_3_ mediated by Ni_2_P NPs was previously reported by the group and was shown to be exothermic to give **INT1**.^[82]^ Subsequent steps involve the interaction between **INT1** with another H_2_O molecule and the substrate at the NiP_2_ surface. This orientation allowed the transfer of two H atoms from the H_2_O molecule and the BH_3_ moiety in **INT1** to the C≡N group, respectively, to form benzylamine. A second transfer of two H atoms from the ‐BH_2_(OH) moiety and another H_2_O molecule to the C=N moiety represented the kinetic key step (3.17 eV) and resulted in the formation of benzylamine as the product.

**Scheme 36:**
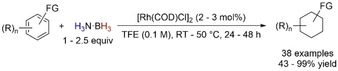
Nonclassical TH of benzene derivatives and heteroarenes mediated by heterogeneous Rh complexes.

Very recently, Glorius and co‐workers reported the TH of benzene derivatives and heteroarenes using H_3_N⋅BH_3_ mediated by [{Rh(cod)(μ‐Cl)_2_}].^[83]^ Moderate to excellent yields were achieved along with good diastereomeric ratio for numerous substrates (Scheme 36). Optimization found that the reaction performed best in fluorinated alcohols, with TFE (2,2,2‐trifluoroethanol) giving the best yield and *d.r*. values; reactions performed in hexane, THF and EtOH resulting in no yield of product. The formation of a black suspension over the course of the reaction indicated the involvement of heterogeneous complexes, which was supported by Hg drop test experiment resulting in no product formation. In addition, Rh nanoparticles (60–100 nm), boron clusters, and aluminum impurities were observed by SEM analysis of the black suspension, supporting a heterogenous mediated catalysis. Probing the reaction further, the average deuterium incorporation into the model substrate (*tert*‐butyldimethyl(*p*‐tolyloxy)silane) using different deuterated H(D)_3_N⋅BH(D)_3_ and TFE indicated that the protic hydrogen was from the solvent and not the NH_3_ counterpart. Furthermore, the reaction was also successful using Me_3_N⋅BH_3_ or HBpin as the boron source. To test whether hydrogenation played a role in the mechanism, the reaction using the model substrate was performed under 1 bar H_2_ without any H_3_N⋅BH_3_ and gave no conversion to the desired product even at 2 bar H_2_. Moreover, letting [{Rh(cod)(μ‐Cl)_2_}] react with H_3_N⋅BH_3_ for ≈3 h then adding the substrate under 1 bar H_2_ resulted in only 8 % conversion, suggesting that H_2_ is deleterious to the reaction. Cumulatively, these experiments indicated a nonclassical TH mechanism in operation mediated by Rh nanoparticles.

## 5 Hydrogenation Reactions

To conclude, in this section we examine examples of hydrogenation using amine–boranes. This route differs from the classical and nonclassical TH reactions presented so far, because the real reducing agent is the H_2_ released in situ. When screening the literature, we observed a paucity of hydrogenation reaction using amine–boranes in homogeneous catalytic systems (Section 3.1).^[37]^ We rationalize this finding, as differentiating whether a homogeneous system is undergoing classical TH or hydrogenation is not trivial. However, we cannot be certain that there have not been examples of the use of alkene traps to monitor gas release in investigations into the DHC of amine–boranes—most literature on this area of chemistry has monitored the direct release of H_2_.^[12]^ What has been reported is the use of cyclohexene to trap H_2_N=BH_2_, with the formation of Cy_2_BNH_2_ as the product but no mention of the formation of cyclohexane.^[84]^

Experimental control reactions can help elucidate whether TH or hydrogenation is occurring. Primarily if no reduction occurs in a homogeneous system when the reaction is performed with H_2_ instead of amine–boranes—this indicates classical TH. If reduction is observed but at a different rate to that observed using amine–borane, then it would also indicate a classical TH process. However, if reduction occurs in the system with H_2_ at the same rate as that using amine–boranes, then the identity of the mechanism is ambiguous and computational insight could be helpful.

The key question in Section 3 is whether the amine–borane's role is specific to forming the active catalytic species to mediate the reduction process as well as providing the hydrogen source? The complication in answering this question is that a common catalytic species is often associated with both classical TH and hydrogenation pathways. However, if the direct release of H_2_ from the amine–borane results in the formation of the active species, then the role of amine–borane is no different to just using H_2_ in the reaction and therefore we classify this as standard hydrogenation.

In contrast, we find that a plethora of examples using heterogeneous catalysts have been reported,^[85]^ and we highlight only those which present productive mechanistic studies for the understanding of the reaction.

### 5.1 Heterogeneous Hydrogenation Reactions with Amine–Boranes

A notable example of a hydrogenation reaction performed with amine–boranes was reported by Manners and co‐workers, who analyzed the formation of catalytically active Rh colloids when reacting [{Rh(cod)(μ‐Cl)_2_}] with H_3_N⋅BH_3_.^[86]^ The new system was able to dehydrogenate H_3_N⋅BH_3_ and sequentially hydrogenate cyclohexene with molecular H_2_ in a closed vessel. When the reaction was performed in an open vessel, no alkene reduction was observed, clearly demonstrating the direct hydrogen addition was taking place in this transformation. Further studies from the same research group on heterogeneous hydrogenation showed that the air‐stable Rh/Al_2_O_3_ system in the presence of Me_2_HN⋅BH_3_ could perform the reduction of alkenes without external H_2_.^[87]^ However, these reactions were still performed in closed vessels and no further evidence of indirect hydrogen transfer was furnished.

Nanoparticulate systems (NPs) based on different metals, alloys, and sizes have been developed and tested in the catalytic reduction of nitriles and nitroarenes, for example, Pd@MIL‐101, Pd NPs enclosed in a mesoporous MOF,^[88]^ and g‐Cu_36_Ni_64_, CuNi NPs grafted on graphite.^[89]^ When H_3_N⋅BH_3_ was simply replaced with H_2_, comparable yields could be found, indicating a clear involvement of the gaseous source. Moreover, tests conducted with open vessels gave lower conversion (<20 %) than experiments with higher pressurized closed vessels, also highlighting that gas evolution and solubility is paramount for the reduction to occur.

**Figure 1:**
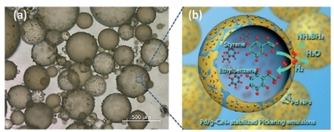
a) Light microscopy image of Pickering emulsions; b) schematic representation of H_3_N⋅BH_3_ DHC/styrene hydrogenation by the Pickering emulsions. Reproduced with permission.

Xu and co‐workers elegantly described a tandem dehydrogenation/hydrogenation of alkenes by H_3_N⋅BH_3_ using Pickering emulsions,^[90]^ which are emulsions stabilized by solid particles instead of surfactants. The authors chose Pd NPs coated onto g‐C_3_N_4_ and carefully analyzed the efficiency and behavior of these microreactors (Figure 1). Importantly, when H_3_N⋅BH_3_ was replaced with gaseous H_2_, low reactivity was observed mainly due to mass transfer effects from the gas to the liquid phase. Moreover, limited emulsification could decrease the reaction efficiency and stirring was found to be beneficial to increase interface area (H_2_ interaction with Pd NPs). These control reactions showed that the Pickering emulsions also function as transient H_2_ storage materials, with potential chemisorbed gas onto Pd and/or formation of gas microbubbles. More importantly, these results highlight that simple interchange between H_3_N⋅BH_3_ and H_2_ might not be sufficient to define the nature of the reduction mechanism, while gas solubility and mass transfer effects need to be considered and tested.

## 6 Summary and Outlook

In this Review we have examined the use of amine–boranes as TH agents. We have carefully analyzed and classified the reduction reactions following three major mechanistic pathways; 1) classical TH, 2) nonclassical TH or solvolysis, and (*3*) hydrogenation with amine–boranes. In each of these contexts we have defined the role of the amine–borane species as the reducing agent and/or precatalyst activator. We have further highlighted the major types of amine–borane derivatives, such as H_3_N⋅BH_3_, more elaborate amine–boranes, and MAB, and categorized these according to their role in reduction reactions.

We have examined the experimental and theoretical characterization techniques which have been used to allow such a mechanistic portrayal. The leading actor is a simple “H_2_ test”, which allows the initial classification of TH versus hydrogenation. However, this experiment is underrated, and it is apparent that this simple test is not used routinely. Moreover, it is crucial to study the role of the solvent to identify nonclassical TH, which is still ill‐defined. The lack of differentiation between solvent‐mediated protonolysis and amine‐mediated protonolysis thereby places some reactions along the continuum between classical and solvolysis‐mediated TH. The indisputable leading techniques are isotope labeling experiments and kinetic analysis for uncatalyzed and catalyzed TH reactions that can allow differentiation between stepwise versus concerted routes.

Each class of TH presented carries its own merits. There is not a single best route but a plethora of different options that can be selected in order to meet the user's requirements. As described, classical TH reactions have proven to be an efficient method to access selectively deuterate substrates with relatively cheap and easy‐to‐handle amine–boranes. Solvolysis TH reactions further allow the introduction of benign and green solvents, whilst classical hydrogenation reactions using amine–boranes allow the use of a very precise quantity of an easy‐to‐handle “drop‐in” source of H_2_ gas and are still the best way to reduce challenging substrates, for example, hindered alkenes. As stated, the area of amine–borane DHC is a buoyant one, but there are virtually no papers which clearly set out to undertake the type of consecutive dual catalysis that is necessary to dehydrogenate an amine–borane then use the H_2_ released to reduce an unsaturated bond, particularly in the field of homogeneous catalysis. This presents a unique opportunity: With a well‐defined catalyst that can undertake these dual roles and with full mechanistic understanding, researchers could be in a strong position to develop further divergent, asynchronous reactions.

We have highlighted that the stepwise transfer hydrogenation mechanism is kinetically and thermodynamically more favorable than dehydrogenation of amine–boranes when homogeneous systems are used. Vice versa, a predominant hydrogenation mechanism is in place when reduction reactions are performed with heterogeneous catalysts. It would be useful to be able to pinpoint why these differences exist: Does it depend on the rate of dehydrogenation versus the rate of stepwise B‐H/N‐H activation? Does this effect depend on the substrate affinity of the catalyst (e.g. better substrate affinity for styrene) versus amine–borane dehydrogenated side‐products? In highlighting this, we hope to fuel discussion and research into this area.

As discussed, most TH substrates are azoarenes and unsaturated hydrocarbons; strikingly few examples of small‐molecule activation have been reported. Rauchfuss et al.^[91]^ have undertaken a highly relevant study on the reduction of O_2_ to H_2_O, a key transformation in fuel cell research. Although not the focus of their study, the authors do report on the ability of H_3_N⋅BH_3_, along with other hydrogen donors, to undertake the reaction in the presence of their Ir catalyst. Beyond this, it appears that the literature is very limited, with only one example of CO_2_ reduction reported by Stephan (Section 3.2) along with a theoretical study of N_2_ reduction on a Ta surface from Paul.^[66]^ It is evident that the TH of small molecules is an area of unmet need: can small molecules such as N_2_, NO_2_, and N_2_O undergo activation and reduction using an amine borane in a laboratory setting and what is the mechanism of these processes? Can we expand on the chemistry of TH of CO_2_ and O_2_, develop new catalysts, and obtain deeper mechanistic understanding?

Another area that is underdeveloped is that of enzymatic TH. Many of the examples reported are deracemizations in the presence of H_3_N⋅BH_3_ and an oxidase.^[92]^ However, it could be argued that the substrates presented are more challenging, or at least more complex, than those tackled using transition metal catalysis. Although mechanistic elucidation is likely to be demanding, a certain level of detail is needed to clearly understand the precise nature of the bond‐breaking and bond‐making process in biological media; the latter aspect is a highly attractive feature of enzymatic chemistry, harmonizing with the benign nature of a TH agent such as H_3_N⋅BH_3_.

Finally, although we have covered several different amine–boranes and their role in TH, great opportunities must exist beyond these standard hydrogen sources. Indeed, elegant studies into the hydrogen‐release properties of amino complex borane encapsulated in metal organic frameworks (MOFs),^[93]^ ethyldiaminoboranes (EDABs),^[94]^ and metal ethyldiaminoboranes (MEDABs)^[95]^ and theoretical studies into boron nitride nanotubes (BNNTs)^[96]^ have been undertaken, but detailed synthetic investigations into their ability to reduce classes of substrate are lacking. Although the TH reagents may not be suitable over a broad spectrum of substrates, they do present an opportunity to probe the extent of reactivity and functional group tolerance, which may allow key or unique targets to be met. As an example, the structures of these unusual amine boranes may lend themselves well to selective reduction of targeted sets of double bonds in a multiply double‐bonded system, for example, in terpene feedstocks.

In conclusion, TH presents great opportunities in mechanistic studies, organic synthesis, and catalyst design. We have identified a plethora of variables associated with TH reactions from: 1) complex substrates through to traditionally inert small molecules, 2) catalyst design ranging from homogeneous, heterogeneous, enzymatic, or even catalyst‐free transformations, 3) the variability of the TH reagent itself, with many possible reagents still to undergo comprehensive testing and opportunities for regio‐ and stereoselectivity, and 4) the nuanced means by which TH takes place that fall into three broad categories. All factors combined indicate that amine–borane‐mediated TH is an area set for growth and likely to be a rich topic of research for years to come.

## Conflict of interest

The authors declare no conflict of interest.

## Biographical Information

*Samantha Lau received her PhD from Imperial College London in 2018 supervised by Dr. Mark Crimmin (ICL) and Dr. Ian Casely (Johnson Matthey). She then started her first postdoctoral position under Dr. Adrian Chaplin at University of Warwick, working on rhodium pincer complexes. In 2019 she began her current postdoctoral position with Dr. Ruth Webster at University of Bath, finally getting her hands on some base metal catalysis*.

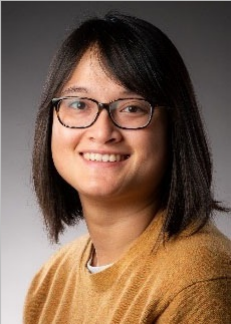

## Biographical Information

*Danila Gasperini studied at the Università di Bologna and Université Pierre et Marie Curie‐UPMC Paris, investigating Pd cross‐couplings with Dr. Julie Oble and Prof. Giovanni Poli (IPCM). In 2014 she moved to University of St Andrews to start her PhD with Prof. Steven Nolan and Prof. Andrew Smith on Au–NHC complexes and their reactivity in catalytic functionalization of alkynes. In 2018 she joined Dr. Ruth Webster's lab at University of Bath, focusing on Fe‐catalyzed transformations of p‐block elements*.

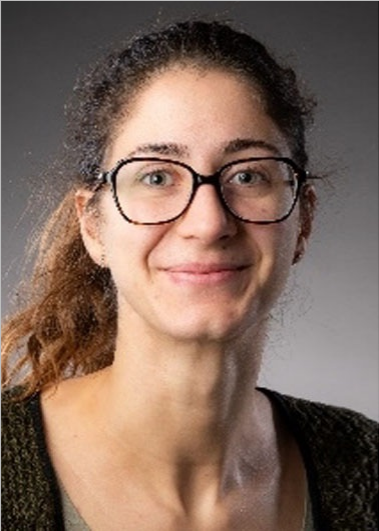

## Biographical Information

*Ruth Webster obtained her undergraduate degree from the University of Strathclyde and her PhD (2011) from the University of Bristol, under the supervision of Prof. Robin Bedford. Following a Commonwealth Postdoctoral Fellowship at the University of British Columbia in the group of Prof. Laurel Schafer, Ruth returned to the UK in 2012, where she is currently an EPSRC Early Career Fellow in Catalysis at the University of Bath*.

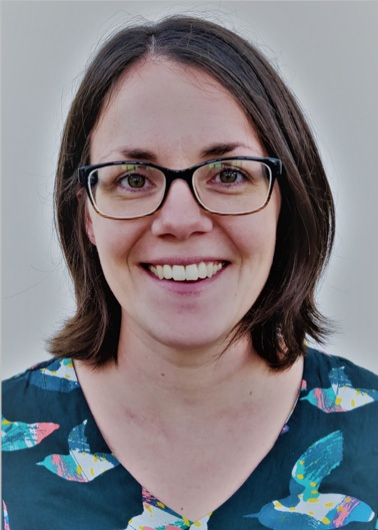

## Supporting information

As a service to our authors and readers, this journal provides supporting information supplied by the authors. Such materials are peer reviewed and may be re‐organized for online delivery, but are not copy‐edited or typeset. Technical support issues arising from supporting information (other than missing files) should be addressed to the authors.

Supplementary
