# Supplementary material for: Amine–Boranes as Transfer Hydrogenation and Hydrogenation Reagents: A Mechanistic Perspective
Source: Angew Chem Int Ed Engl. 2021 Feb 25;60(26):14272–94. doi: 10.1002/anie.202010835 (PMC8248159; doi:10.1002/anie.202010835)
Supplement: Supplementary file 1 — Supplementary [file ANIE-60-14272-s001.pdf]

Angewandte Chemie
Eine Zeitschrift der Gesellschaft Deutscher Chemiker

Supporting Information

Amine–Boranes as Transfer Hydrogenation and Hydrogenation Reagents: A Mechanistic Perspective

*Samantha Lau, Danila Gasperini, and Ruth L. Webster**

anie_202010835_sm_miscellaneous_information.pdf

Due to page constraints, the following examples of transfer hydrogenation (TH) reactions with amine-boranes could not appear in the main body of the review. However, we believe that these should be referred to for rounded understanding of the topic.

2. Catalyst-free Classical TH of Preactivated Substrates

Early examples of using amine-boranes as reducing reagents have been reported since the 1980s by Andrews and Crawford on the reduction of aldehydes and ketones.[1] Optimization reactions found the reduction worked well in both protic and aprotic solvents. However, mechanistic details were not described. Furthermore, asymmetric reduction of aromatic ketones using optically active alkoxy amine-borane complexes as the reducing agent were explored by Hirao and co-workers in 1981, with good conversion to the secondary alcohol but with modest optical purity.[2]

In 2014, Kinjo and co-workers reported the synthesis and isolation of a novel C_4 cumulene derivative.[3] In order to test the reactivity of this compound, it was reacted in THF at 60 °C with 2 equiv. of $H_3N{\cdot}BH_3$ for 1 h to afford the reduction of the central double bond (Scheme S1a). This represented the first metal-free TH of a non-polar bond with $H_3N{\cdot}BH_3$. Deuterium labelling experiments using $D_3N{\cdot}BD_3$ showed deuterium incorporation at both carbon positions. Computational calculations indicated a smaller energy gap between the HOMO of the cumulene and the LUMO of $H_3N{\cdot}BH_3$ (ΔE = 5.36 eV) compared to the HOMO of $H_3N{\cdot}BH_3$ and LUMO of cumulene (ΔE = 9.21 eV) (Scheme S1b). This might indicate the mechanism involved the initial deprotonation of the N–H rather than transfer of the B–H to carbon. However, they stipulated a concerted double hydrogen transfer could also be operating. Additional DKIE experiments using $D_3N{\cdot}BH_3$ and $H_3N{\cdot}BD_3$ could be insightful into deciphering if a concerted or more stepwise addition is occurring similar to what Berke and co-workers found in their investigations (*vide* Scheme 2 in the manuscript).

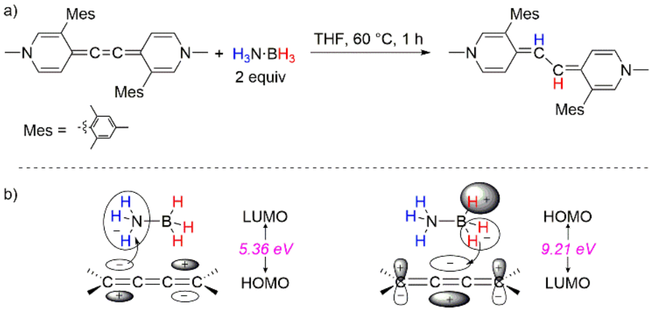

Scheme S1. a) TH of cumulene derivative; b) HOMO-LUMO pairs of the substrate and $H_3N{\cdot}BH_3$.

3. Catalyzed Classical TH Reactions

Waterman and co-workers expanded on the catalysis of group 4 with the TH of olefins using [Zr(N_3N)NMe_2] (N_3N = $N(CH_2CH_2NSiMe_2CH_2)_3$).[4] Amine-boranes (RR'HN·BH_3, R = R' = Me; R = *t*Bu, R' = H) were generally not a good reagent for this reaction, with competitive hydroboration products formed along with reduced alkanes. Reactions where the substrate was subjected to 1 atm of H_2 under catalytic conditions did not show significant conversions. However, $H_3N{\cdot}BH_3$ allowed the suppression of side-reactions, possibly due to its fast dehydrocoupling, and quantitative reductions to be obtained in THF-d_8 or benzene-d_6 at 65 °C. Waterman further expanded on the TH of olefins with $H_3N{\cdot}BH_3$, using Cp*Co(CO)I_2.[5]

N-heterocyclic silylene (NHSi) Mn(II) complexes were also active pre-catalysts for the semi-TH of alkynes, however this procedure led preferentially to *E*-alkenes.[6] No conversion occurred in an H_2 atmosphere, and the author suggested that a [Mn–H] intermediate could form in the reaction media claiming that a vibration mode of the metal-hydride bond was detected when following the reaction by IR spectroscopy.

4. Solvolysis of Amine Boranes in non-classical TH Reactions

4.2 Homogeneous mediated solvolysis

A study into the reduction of alkynes using a PNP Mn pincer complex was reported recently by El-Sepelgy, Azofra and co-workers (Scheme S2).[7] Optimization and control reactions determined that: MeOH was the best solvent, using

$MnCl_2$ gave low conversion of alkyne and poor stereoselectivity, performing the reaction using 2 mol% catalyst, 2.5 mol% $H_3N{\cdot}BH_3$ under 10 bar of H_2 led to trace amount of desired product, and similarly using formic acid as the hydrogen source lead to trace amount of product. These experiments were a good indication that $H_3N{\cdot}BH_3$ is necessary for the success of the reaction not only to activate the Mn pre-catalyst but also as the hydrogen source to regenerate the catalyst.

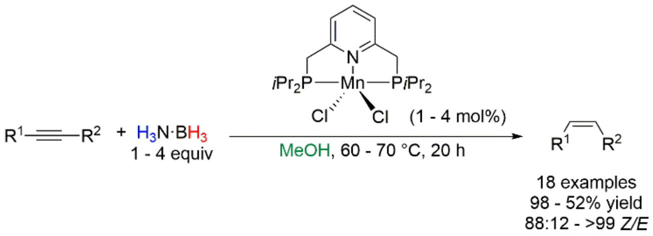

SchemeS2. Stereo- and chemoselective hydrogenation of alkenes mediated by PNP Mn pincer complex

A similar reaction mechanism to the one postulated by Liu, Luo and co-workers[8] was proposed involving the generation of a [Mn–H] complex as the active species. DFT calculations were performed to confirm the plausibility of the reaction pathway. A triplet spin state route was indicated by the calculation. For the calculated pathway the RDS was found to be proton transfer from the MeOH to the alkenyl-Mo species at $\Delta G^{\ddagger}_{(323)} = 25.6$ kcal mol^{-1}. An alternative pathway with $H_3N{\cdot}BH_3$ as the hydride *and* proton source was found to be higher in energy ($\Delta G^{\ddagger}_{(323)} = 37.1$ kcal mol^{-1}) and therefore discounted as a potential pathway when the reaction was performed in MeOH. Additional deuterium labelling experiments could have provided more evidence supporting this mechanism with deuterium incorporation expected in the product if using MeOD and no deuterium incorporation if using $D_3N{\cdot}BH_3$. Furthermore, DKIE experiments should distinctly show an effect when using MeOD if this was the RDS of the mechanism.

4.3 Heterogeneous mediated solvolysis

In 2020, Balaraman and co-workers fabricated cobalt nanoparticles on nitrogen-doped graphene Co@NRGs to semi-TH of alkynes in MeOH at 80 °C.[9] The authors recognized the solvolysis mechanism in action by labelling studies; using CD_3OD they observed 90% deuterium incorporation into cis-stilbene, although only in one of the two positions which highlights that the solvent acts only as protic source and that proto-demetalation of Co-alkenyl intermediate is solvent mediated (see Scheme 31 in the manuscript). Negligible reduction was observed using molecular H_2. Catalyst recyclability experiments shown the stability of the system, and most notably XRD, TEM and XPS analysis of the reused catalysts revealed that Co^{2+} valence state is retained, and presence of graphite, Co and CoO are comparable to those present in the fresh catalysts.

[1] a) G. C. Andrews, T. C. Crawford, *Tetrahedron Lett.* **1980**, *21*, 693-696; b) G. C. Andrews, *Tetrahedron Lett.* **1980**, *21*, 697-700.

[2] A. Hirao, S. Itsuno, S. Nakahama, N. Yamazaki, *J. Chem. Soc., Chem. Commun.* **1981**, 315-317.

[3] D. Wu, Y. Li, R. Ganguly, R. Kinjo, *Chem. Commun.* **2014**, *50*, 12378-12381.

[4] K. A. Erickson, J. P. W. Stelmach, N. T. Mucha, R. Waterman, *Organometallics* **2015**, *34*, 4693-4699.

[5] J. K. Pagano, J. P. W. Stelmach, R. Waterman, *Dalton Trans.* **2015**, *44*, 12074-12077.

[6] Y.-P. Zhou, Z. Mo, M.-P. Luecke, M. Driess, *Chem. Eur. J.* **2018**, *24*, 4780-4784.

[7] A. Brzozowska, L. M. Azofra, V. Zubar, I. Atodiresei, L. Cavallo, M. Rueping, O. El-Sepelgy, *ACS Catal.* **2018**, *8*, 4103-4109.

[8] S. Fu, N.-Y. Chen, X. Liu, Z. Shao, S.-P. Luo, Q. Liu, *J. Am. Chem. Soc.* **2016**, *138*, 8588-8594.

[9] G. Jaiswal, V. G. Landge, M. Subaramanian, R. G. Kadam, R. Zbořil, M. B. Gawande, E. Balaraman, *ACS Sustainable Chem. Eng.* **2020**, *8*, 11058-11068.
